# Proteomic analysis of proteins expressing in regions of rat brain by a combination of SDS-PAGE with nano-liquid chromatography-quadrupole-time of flight tandem mass spectrometry

**DOI:** 10.1186/1477-5956-8-41

**Published:** 2010-07-27

**Authors:** Tomoki Katagiri, Naoya Hatano, Masamune Aihara, Hiroo Kawano, Mariko Okamoto, Ying Liu, Tomonori Izumi, Tsuyoshi Maekawa, Shoji Nakamura, Tokuhiro Ishihara, Mutsunori Shirai, Yoichi Mizukami

**Affiliations:** 1 Center for Gene Research, Yamaguchi University, Yamaguchi, 755-8505, Japan; 2 Department of Rare Sugar Research Center, Kagawa University, Kagawa, 761-0793, Japan; 3 First Department of Pathology, Yamaguchi University Graduate School of Medicine, Yamaguchi, 755-8505, Japan; 4 Department of Neuroscience Yamaguchi University Graduate School of Medicine, Yamaguchi, 755-8505, Japan; 5 Department of Stress and Bio-response Medicine, Yamaguchi University Graduate School of Medicine, Yamaguchi, 755-8505, Japan; 6 Department of Microbiology and Immunology, Yamaguchi University Graduate School of Medicine, Yamaguchi, 755-8505, Japan

## Abstract

**Background:**

Most biological functions controlled by the brain and their related disorders are closely associated with activation in specific regions of the brain. Neuroproteomics has been applied to the analysis of whole brain, and the general pattern of protein expression in all regions has been elucidated. However, the comprehensive proteome of each brain region remains unclear.

**Results:**

In this study, we carried out comparative proteomics of six regions of the adult rat brain: thalamus, hippocampus, frontal cortex, parietal cortex, occipital cortex, and amygdala using semi-quantitative analysis by Mascot Score of the identified proteins. In order to identify efficiently the proteins that are present in the brain, the proteins were separated by a combination of SDS-PAGE on a C18 column-equipped nano-liquid chromatograph, and analyzed by quadrupole-time of flight-tandem-mass spectrometry. The proteomic data show 2,909 peptides in the rat brain, with more than 200 identified as region-abundant proteins by semi-quantitative analysis. The regions containing the identified proteins are membrane (20.0%), cytoplasm (19.5%), mitochondrion (17.1%), cytoskeleton (8.2%), nucleus (4.7%), extracellular region (3.3%), and other (18.0%). Of the identified proteins, the expressions of glial fibrillary acidic protein, GABA transporter 3, Septin 5, heat shock protein 90, synaptotagmin, heat shock protein 70, and pyruvate kinase were confirmed by immunoblotting. We examined the distributions in rat brain of GABA transporter 3, glial fibrillary acidic protein, and heat shock protein 70 by immunohistochemistry, and found that the proteins are localized around the regions observed by proteomic analysis and immunoblotting. IPA analysis indicates that pathways closely related to the biological functions of each region may be activated in rat brain.

**Conclusions:**

These observations indicate that proteomics in each region of adult rat brain may provide a novel way to elucidate biological actions associated with the activation of regions of the brain.

## Background

The mammalian central nervous system regulates higher biological actions such as feelings and behaviors, which are known to associate with the activation of specific regions of brain as determined by positron-emission topography and MRI techniques [1-3]. A characterization of all components expressed in each region is essential to understand the mechanisms of higher actions and their functional properties. Recently, methods for the comprehensive analyses of gene expression such as DNA array and serial analysis gene expression have been developed in addition to the disclosure of genomic sequence information [4-7]. These applications have provided much information about gene expression in the brain [8-13]. A comprehensive account of the products of gene expression through protein profiling is needed even more than genome information to fully understand higher biological actions.

Neuroproteomics has been applied to the analysis of whole brain, and the general pattern of protein expression in all regions has been elucidated [14-16]. In specific regions, such as the hippocampus, thalamus, and striatum, the relationships between protein stimulations or diseases have been examined by proteomic analysis [17-20]. However, the comprehensive proteome of each brain region remains unclear despite the attention paid to the biological functions of each region, although comprehensive investigations of gene expression in each region of the brain have been undertaken using DNA array and in situ hybridization techniques [8].

We have undertaken proteomic analysis using a variety of protocols according to the research targets, such as the combination of two-dimensional electrophoresis with matrix-assisted laser desorption/ionization time-of-flight mass spectrometry and nano-liquid chromatography (LC)-quadrupole-time of flight (Q-TOF)-tandem mass spectrometry (MS/MS) after column concentration [21-27]. Here, we have divided the rat brain into six regions, thalamus, hippocampus, frontal cortex, parietal cortex, occipital cortex, and amygdala, and compared the proteome of each region by a combination of sodium dodecyl sulfate (SDS)-polyacrylamide gel electrophoresis (PAGE) and nano-LC-Q-TOF-MS/MS. We have identified a total of 2,909 peptides in all regions of the rat brain, and found proteins specifically expressed in each region of brain: 63 proteins in the thalamus, 38 in the hippocampus, 14 in the frontal cortex, 66 in the parietal cortex, 24 in the occipital cortex, and 36 in the amygdala by semi-quantitative analysis.

## Results

### Identification of proteins in each region of rat brain

**Figure 1:**
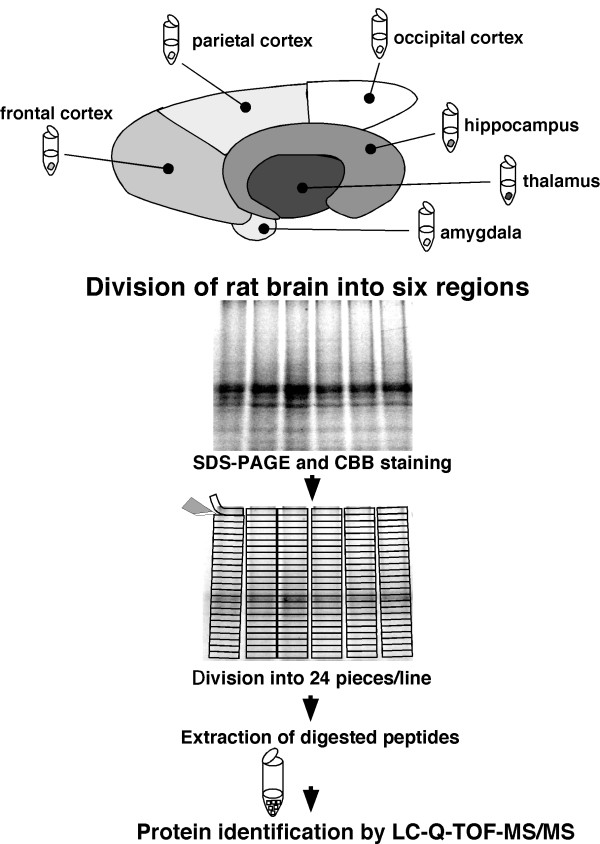
**Flow diagram of the experimental design**. Rat brains were divided into six regions: thalamus, hippocampus, frontal cortex, parietal cortex, occipital cortex, and amygdala. The divided samples were lysed in lysis buffer containing SDS, and subjected to SDS-PAGE with Coomassie Brilliant Blue staining. The gel lane was divided into 24 slices, and the slices were pre-treated by in-gel trypsin digestion. The amino acid sequences of all detected proteins were determined by nano-LC-Q-TOF-MS/MS.

**Figure 2:**
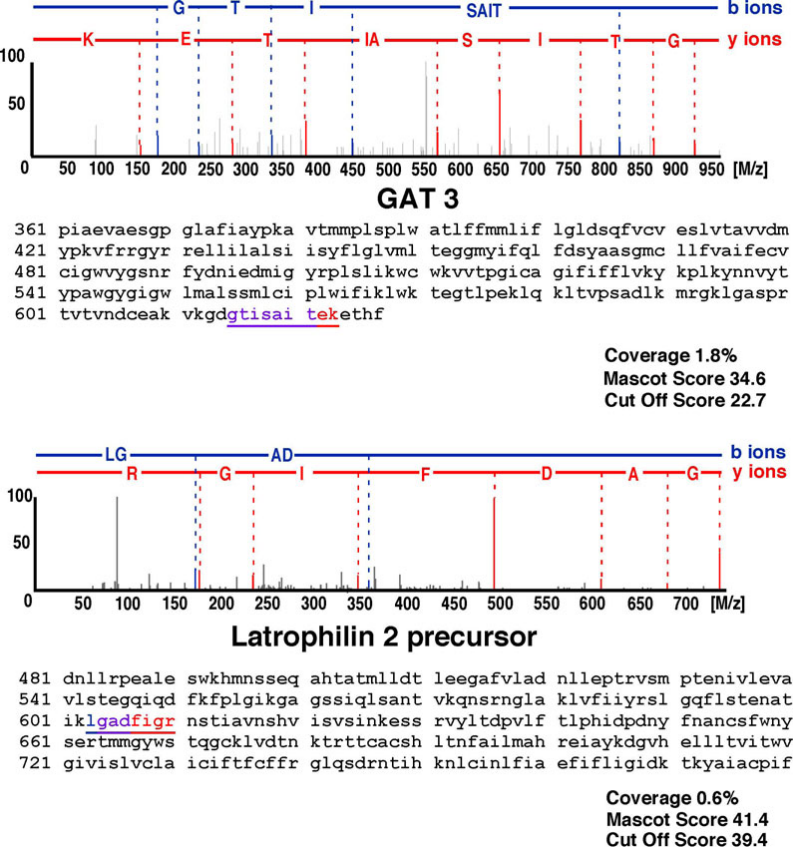
**Representative MS/MS spectra of proteins identified in rat brain**. The MS/MS spectrum for the peptide derived from GAT 3 (A), identified only in the thalamus of the rat brain, is shown; the amino acid sequence GTISAITEK deduced from the 5 b type ions (blue) and 8 y type ions (red) were assigned by Mascot search (upper panel). The identified sequence within the entire amino acid sequence of GAT 3 is indicated by the underline (lower panel). The MS/MS spectrum for the peptide derived from the Latrophilin 2 precursor (B), identified only in the parietal cortex of the rat brain, is shown; the amino acid sequence GADFIGR deduced from the 2 b type ions (blue) and 7 y type ions (red) were assigned by Mascot search (upper panel). The identified sequence within the entire amino acid sequence of Latrophilin 2 is indicated by the underline (lower panel).

**Figure 3:**
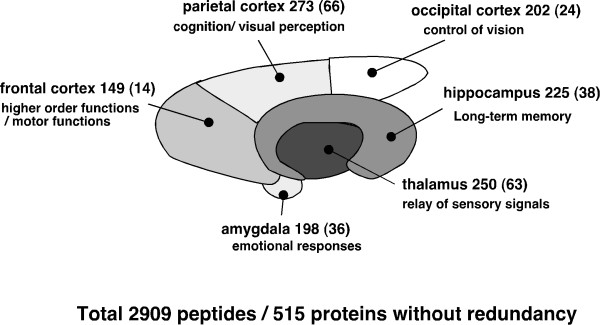
**Schematic representation of proteins identified in six regions of rat brain**. In total, 2,909 peptides including redundant peptides were identified by nano-LC-Q-TOF-MS/MS in all regions of the brain, leaving a total of 515 proteins. By region, 250 proteins were identified in the thalamus, 225 in the hippocampus, 149 in the frontal cortex, 273 in the parietal cortex, 202 in the occipital cortex, and 198 in the amygdala without redundancy. Sixty-three proteins in the thalamus, 38 in the hippocampus, 14 in the frontal cortex, 66 the in parietal cortex, 24 in the occipital cortex, and 36 in the amygdala were found in only that region of the brain.

**Figure 4:**
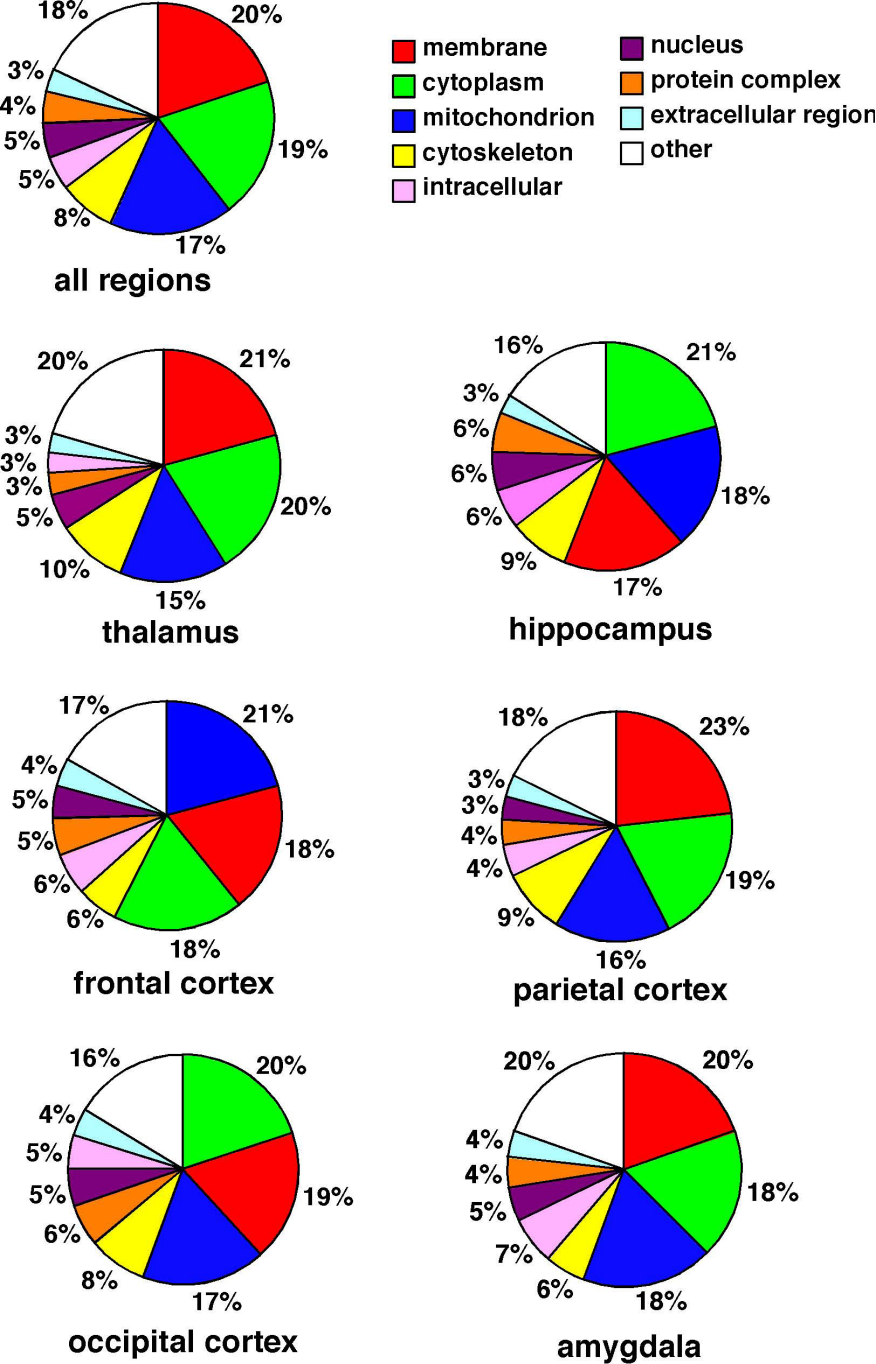
**Intracellular localization of proteins identified by Q-TOF-MS/MS in rat brain**. The intracellular localizations of all proteins identified by nano-LC-Q-TOF-MS/MS were classified based on the component section in NCBI Entrez Gene.

To identify proteins expressing in each region of rat brain, the removed brains were divided into six regions, thalamus, hippocampus, frontal cortex, parietal cortex, occipital cortex, and amygdala, according to the atlas of Paxinos and Watson. To test the efficiency of protein extraction by lysis buffer, we determined the protein concentrations extracted by lysis buffers included Triton X-100, CHAPS, NP-40, or SDS. The extracted proteins were determined by EZQ protein quantification kit (Molecular Probe), which is able to determine the protein concentrations in the presence of the detergents. The proteins concentration in the solution extracted by SDS sample buffer was significantly high compared with those in other lysis buffers (data not shown). The proteins in each region were extracted by lysis buffer including SDS, and separated by electrophoresis. Each gel lane was sliced into 24 pieces, and the proteins extracted from the gel pieces were applied to nano-LC-Q-TOF-MS/MS and analyzed by the Mascot search engine to identify amino acid sequences (Fig. 1). All together in all brain regions, 2,909 redundant peptides were assigned, and 515 proteins were identified with more than 95% confidence based on the amino acid sequences deduced from the MS/MS peaks (Additional file 1, Table S1). The proteins assigned by a single peptide were confirmed manually. Representative MS/MS spectra of the identified proteins GABA transporter 3 (GAT 3) and Latrophilin 2 are shown in Fig. 2. GAT 3 was identified based on the amino acid sequence GTISAITEK deduced by both b type ions and y type ions on the MS/MS spectrum, the sequence of which corresponds to 1.8% of the whole sequence. For the Latrophilin 2 precursor, the amino acid sequence deduced from the MS spectrum is LGADFIGR, which covers 0.6% of the full sequence. The peaks used for the identification of Latrophillin 2 were observed more prominently than other peaks on the spectrum, and the confidence level calculated from the peak data was greater than 95% by Mascot search. The numbers of proteins identified in each region without redundancy were 250, 225,149, 273, 202, and 198 for thalamus, hippocampus, frontal cortex, parietal cortex, occipital cortex, and amygdala, respectively (Fig. 3). Among the identified proteins, the number of region-abundant proteins was 63 in thalamus, 38 in hippocampus, 14 in frontal cortex, 66 in parietal cortex, 24 in occipital cortex, and 36 in amygdala (Fig. 3) by semi-quantitative analysis. Several functional molecules were observed in the specific regions, such as G protein coupled receptors (thyroid stimulating hormone receptor 1 and Latrophilin 2) in the parietal cortex, and olfactory receptors (olfactory receptor family 10 and olfactory Olr 436) in the occipital cortex (Table 1). Next we analyzed the intracellular localization of the identified proteins based on the component section in the NCBI Entrez Gene. In all regions, most proteins localized in the membrane fraction (20.0%), with other proteins classified into cytoplasm (19.5%), mitochondrion (17.1%), cytoskeleton (8.2%), nucleus (4.7%), extracellular region (3.3%), and other (18.0%) (Fig. 4).

**Table 1:** Representative membrane proteins identified in each region of adult rat brain by Q-TOF-MS/MS

NCBI ID	Protein Name	Region	Localization	Function
**Receptor**				

gi|94380294	PREDICTED: similar to Carcinoembryonic antigen-related cell adhesion molecule 1 precursor (Biliary glycoprotein 1) (BGP-1) (Murine hepatitis virus receptor) (MHV-R)	T	ND	
gi|32401457	opsin 5	H, A	ND	G-protein coupled receptor
gi|38259186	adiponectin receptor 1	F	M	fatty acid metabolism
gi|62990176	thyroid stimulating hormone receptor	P	M	G-protein coupled receptor
gi|6912464	latrophilin 2 precursor	P	M	G-protein coupled receptor
gi|52317184	olfactory receptor, family 10, subfamily X, member 1	O	M	olfactory receptor
gi|47577367	olfactory receptor Olr436	O	M	olfactory receptor

**Ion channel**				

gi|6755963	voltage-dependent anion channel 1	T, H, F, P, O, A	M, Mt	voltage-gated ion-selective channel activity

**Na Pump**				

gi|21450321	Na+/K+ -ATPase alpha 3 subunit	T, H, F, P, O, A	ND	
gi|30409956	ATPase, Na+/K+ transporting, alpha 2 polypeptide	T, H, F, P, O	M	sodium:potassium-exchanging ATPase
gi|6978543	ATPase, Na+/K+ transporting, alpha 1 polypeptide	T, F, P, O, A	M	sodium:potassium-exchanging ATPase
gi|6978549	ATPase, Na+/K+ transporting, beta 1 polypeptide	T, H, F, P, O, A	M	sodium:potassium-exchanging ATPase

**Ca Pump**				

gi|62234487	plasma membrane calcium ATPase 1	T, H, P, O	M	calcium-transporting ATPase
gi|48255951	plasma membrane calcium ATPase 2 isoform a	P, O	M	calcium-transporting ATPase

**Proton Pump**				

gi|34856315	PREDICTED: similar to ATPase, H+ transporting, V1 subunit B, isoform 1	T, H, O, A	ND	
gi|62665162	PREDICTED: ATPase, H+ transporting, V0 subunit D isoform 1 (predicted)	T, H, F, P, A	ND	
gi|34869154	PREDICTED: similar to ATPase, H+ transporting, V1 subunit A, isoform 1	T, H, F, P	ND	
gi|12025532	ATPase, H+ transporting, lysosomal V0 subunit a isoform 1	T, F, P	Cy, M, N	hydrogen ion transporter activity
gi|47717102	ATPase, H+ transporting, lysosomal 50/57kDa, V1 subunit H isoform 2	T	Cy, M	hydrogen-transporting ATP synthase activity
gi|19913426	ATPase, H+ transporting, lysosomal 56/58kDa, V1 subunit B1	H, F, O, A	Cy, M, I	hydrogen-transporting ATPase
gi|124244102	ATPase, H+ transporting, lysosomal V0 subunit a isoform 2	P	Cy, Ex, M	

**Transporter**				

gi|78126167	solute carrier family 1 (glial high affinity glutamate transporter), member 2 isoform a	T, H, F, P, O, A	M	glutamate transport
gi|9507115	solute carrier family 1 (glial high affinity glutamate transporter), member 3	P, A	M	glutamate transport
gi|400626	Sodium- and chloride-dependent GABA transporter 3	T	M	neurotransmitter transport

T, thalamus; H, hippocampus; F, frontal cortex; P, parietal cortex; O, occipital cortex; A, amygdala

M, membrane; Ex, extracellular region; Mt, mitochondrion; N, nucleus; Cy, cytoplasm; I, intracellular; ND, not described

### Confirmation of protein expression in each region of rat brain

**Figure 5:**
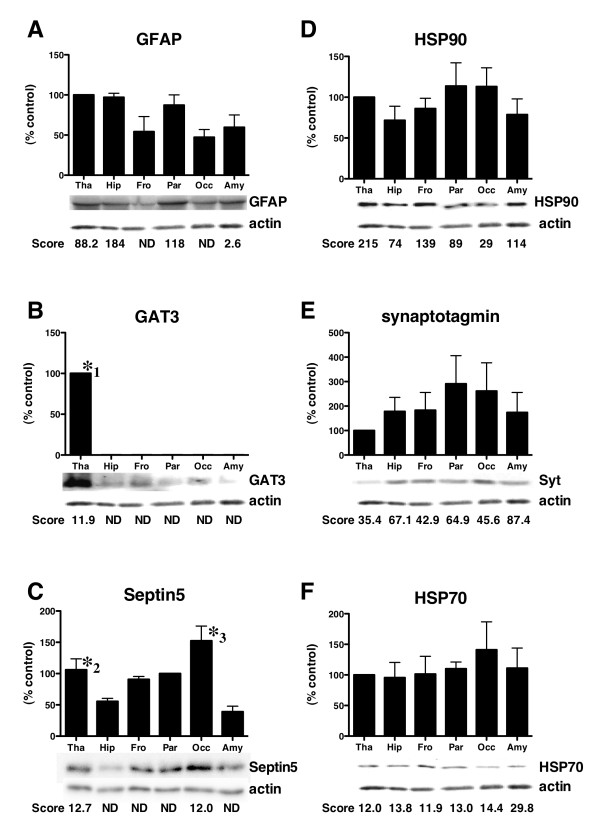
**Immunoblotting analysis of proteins identified in six regions of rat brain**. Rat brains were divided into six regions (thalamus, hippocampus, frontal cortex, parietal cortex, occipital cortex, and amygdala), and were extracted with lysis buffer. The samples (2.0 μg protein) were subjected to immunoblotting with anti-GFAP antibody (A), anti-GAT 3 antibody (B), anti-Septin 5 antibody (C), anti-HSP 90 antibody (D), anti-synaptotagmin antibody (E), and anti-HSP 70 antibody (F). Immunoblotting data using the indicated antibodies are shown in the upper panels; Mascot scores of the proteins determined by Q-TOF-MS/MS are shown in the lower panels. The immunoblotting data are shown as representative blots obtained in independent experiments from 3 rats. Data represent the means ± SE. Statistical significance was determined by ANOVA followed by Bonferroni's test. *1 *p < 0.05 *vs Hip, Fro, Par, Occ, and Amy, *2 *p < 0.05 *vs Amy, *3 *p < 0.05 *Amy and Hip.

**Figure 6:**
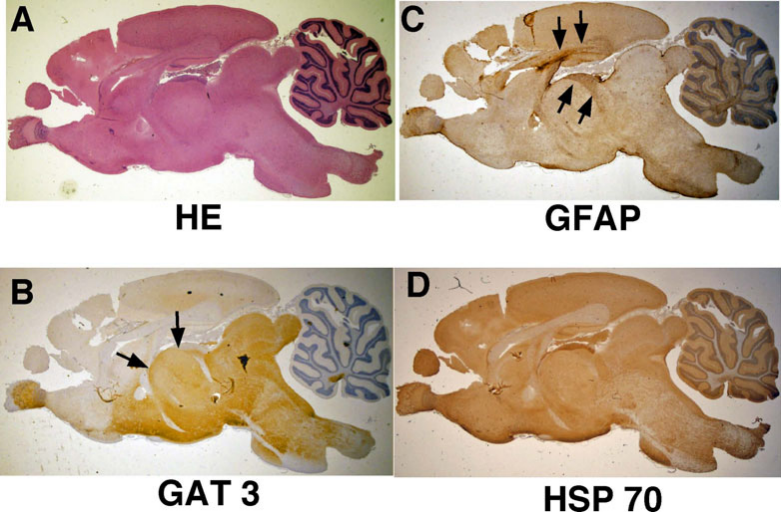
**Immunohistochemical analysis of proteins identified by nano-LC-Q-TOF-MS/MS in rat brain**. Rat brains were removed, fixed with 4% paraformaldehyde, and stained with hematoxylin and eosin (A), anti-GAT 3 antibody (B), anti-GFAP antibody (C), or anti-HSP 70 antibody (D) as described in "Experimental Procedures". The panels show representative photographs from independent experiments from 3 rats. Final magnification × 10.

To confirm the brain distribution of proteins identified in the six regions by Q-TOF-MS/MS, we carried out immunoblotting using specific antibodies, and the results were compared with Mascot scores, since there is a correlation between the protein amounts on the gels and Mascot score [22]. Immunoblotting using the anti-glial fibrillary acidic protein (GFAP) antibody demonstrated that bands were observed in all regions, and the densities were increased in the thalamus and hippocampus, a finding that is almost the same as that obtained using Mascot scores (Fig. 5A). GAT 3 was only detected in the thalamus by immunoblotting, which is completely consistent with the results obtained by MS/MS analysis (Fig. 5B). Septin 5 detected in only Parietal cortex by mass analysis, was mainly observed as single band on the immunoblots in Parietal cortex fraction (Fig. 5C). The small amount of Septin 5 was observed in other regions. The difference of data might be due to the sensitivity between immunoblotting and mass analysis. The anti-heat shock protein (HSP) 90 antibody recognized a protein with an approximate molecular mass of 90 kDa in all regions of the brain on the immunoblots, but the band densities in the parietal cortex and occipital cortex were slightly reduced compared with other regions (Fig. 5D). The Mascot scores for HSP 90 were also low in both regions (Fig. 5D). The amount of synaptotagmin (Fig. 5E), HSP 70 (Fig. 5F), and pyruvate kinase (data not shown) were similar in all regions by immunoblotting using specific antibodies. Consistent with these blotting data, the proteins were detected ubiquitously in all regions with constant values in the Mascot scores (Fig. 5E and 5F). Furthermore, we carried out a distribution analysis with antibodies for GFAP, GAT 3, and HSP 70, useful for immunohistochemistry, to confirm the data obtained by the immunoblotting and MS/MS analysis. Immunohistochemical observations indicated that GAT 3 shows preferential staining in the thalamus, hypothalamus, medulla, and olfactory bulb, consistent with the MASCOT score data (Fig. 6A and 6B). GFAP staining was observed in all regions, and was enhanced in part of the hippocampus and thalamus (Fig. 6A and 6C). HSP 70 was ubiquitously detected in all regions of the brain by immunohistochemistry (Fig. 6A and 6D). These data are mostly consistent with the data obtained by immunoblotting and MS/MS analysis. Together with all of the observations, the data determined by nano-LC-Q-TOF-MS/MS are reliable for the identification of brain proteins.

### IPA analysis in proteins identified in each region of rat brain

**Figure 7:**
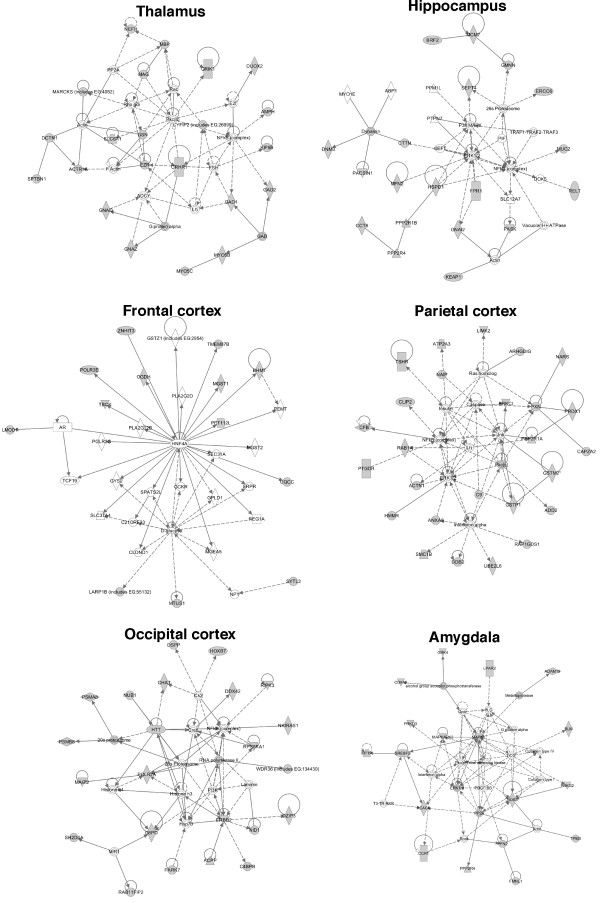
**Network analysis of proteins identified in each region of rat brain by Ingenuity pathway analysis**. The networks were analyzed based on the data of proteins identified in the indicated regions of rat brain. The networks were revealed as circles (genes) and lines (biological relationship). Solid lines mean direct interaction, and dotted lines show indirect interactions between the genes.

We carried out the pathway analysis by IPA analysis using the data of proteins identified in each region of rat brain. The representative networks in each region were shown in Fig.7 with Additional file 2, Table S2 and the canonical pathways were also revealed in Additional file 3, Fig. S1. The pathway through corticotropin releasing hormone receptor was detected in thalamus releasing corticotropin-releasing hormone under stress (Fig.7). In hippocampus, the pathways through ERK1 and S6 kinase were detected n Fig.7 and Additional file 3, Fig. S1, suggesting that the development and functions in nervous system may be activated through growth factors such as BDNF in the region. The pathway related to citrate cycle was observed in frontal cortex and the pathway involving in mitochondria dysfunction in amygdala was shown in Additional file 3, Fig. S1.

## Discussion

We have conducted a comprehensive analysis of the proteins expressing in six regions of rat brain following a proteomic approach using SDS-PAGE and nano-LC-Q-TOF-MS/MS. As a result, we identified 250 proteins in thalamus, 225 in hippocampus, 149 in frontal cortex, 273 in parietal cortex, 202 in occipital cortex, and 198 in amygdala. Furthermore, the localizations of several proteins identified by proteomics were confirmed by immunoblotting and immunohistochemistry using specific antibodies.

Membrane proteins including ion channels, receptors, and ion transporters play important roles in brain functions, with nerve cells in particular utilizing diverse functional proteins on the membrane for receiving, conducting and transmitting signals. In this report, the samples extracted directly by SDS from each region of the brain were separated using SDS-PAGE. The approach has advantages in identifying many proteins including membrane proteins in the brain because the SDS essential for electrophoresis is a strong detergent that lyses most proteins, and can be used directly for the preparation of brain samples, although the resolution cannot be improved. Using this protocol, proteins expressed in the brain were efficiently identified, and over 20% of the identified proteins were deduced to localize in membranes. In hippocampal samples prepared by two-dimensional electrophoresis, over 70% of the identified proteins were cytoplasm-localized, while only 7% were detected as membrane proteins [17].

By comparing the proteins found in each region, proteins closely related to the function of each region have been identified in this study. Of the identified proteins, GAT 3 was found only in the thalamus, and this specific expression was confirmed by immunoblotting. In the mammalian thalamus, GABA is a major inhibitory neurotransmitter, and the GABA transporter mediates GABA uptake into presynaptic terminals to terminate the effects of GABA [28,29]. Consistent with our data, ultrastructural investigations show that GABA transporter 3 is expressed most prominently in the thalamus [8,30]. The immunohistochemical investigation also detected GAT 3 in the olfactory bulb and hypothalamus, and GAT 3 may be expressed except for the regions used in this study [31]. In samples extracted from the occipital cortex, two types of olfactory receptors were specifically identified. Olfactory receptors are understood to function as molecular sensors for odorants [32]. Olfactory receptors are expressed mostly in pyramidal cells of the occipital cortex, which is consistent with our data [33]. Olfactory stimulation results in the activation of the bilateral occipital cortex as detected by positron-emission topography [34]. Improvements in extra-conversion and regional cerebral blood flow might be induced by smell stimulation through the activation of the occipital cortex [35]. The olfactory receptors expressed in the occipital cortex may be involved in biological functions, resulting in smell stimulation. Septin 5 participating in neuronal development was mainly detected in occipital cortex and thalamus. Septin 5 is colocalized in synaptic vesicles with SNARE proteins, and plays a role in neurotransmitter release [36]. However, *Septin 5 *mice showed no changes in synaptic transmission in hippocampus [37]. In our data, the protein amount of Septin 5 in hippocampus was much less than other regions as shown in Fig. 5 and mass analysis, which was consistent with data with *Septin 5*^-/-^mice. In the hippocampal region related to memory, we identified Glyoxalase 1, the localization of which was found to be mostly consistent with that determined by in situ hybridization [8]. The specific expression of Glyoxalase 1, as indicated in this study, suggests the involvement of Glyoxalase 1 in the biological functions of the hippocampus such as memory and long-term potentiation. In fact, Chen et al. indicate that Glyoxalase 1 plays a novel role in Alzheimer's disease and frontotemporal dementia [38].

Network analysis of the proteins expressed in each region of rat brain indicates that these proteins were linked in a pathway. Many of these proteins have associated with amino acid metabolism, molecular transport, and small molecular biochemistry in most regions, which is consistent with the previous observations that neuron activity in the brain was regulated by small molecules such as neurotransmitter and ion transport. In hippocampus playing an important role in long-term memory, the pathways involving in signal transduction such as ERK, NF-kB, S6 kinase, and mTOR were observed by IPA analysis. Long-term depression in hippocampus requires rapid protein synthesis, which is associated with mTOR, ERK, and S6-dependent signaling pathways [39]. Serum- and glucocorticoid-inducible kinase1 increased the acetylation and activation of NF-kB through phosphorylation of p300, and this also leads to the expression of N-methyl-d-aspartate receptor, NR2A and NR2B that is implicated in neuronal plasticity in hippocampus [40]. Dynamic chromatin remodeling in hippocampal neurons are associated with N-methyl-d-aspartate receptor-mediated activation of Bdnf gene promoter 1 [41]. BDNF activates ERK pathway in hippocampus neurons. The pathways shown by IPA analysis based on the data are closely related to the biological functions reported in each region of brain, suggesting that proteomic profiling of regions may be useful for the elucidation of biological functions.

## Conclusions

A total of 2,909 peptides in all regions of the rat brain were identified, and we displayed proteins expressed in each region of brain: 250 proteins in the thalamus, 225 in the hippocampus, 149 in the frontal cortex, 273 in the parietal cortex, 202 in the occipital cortex, and 198 in the amygdala. Of the identified proteins, the expressions of GFAP, GAT3, Septin 5, HSP 90, synaptotagmin, HSP 70, and pyruvate kinase were confirmed by immunoblotting, and localizations of GAT3, GFAP, and HSP 70 were confirmed by immunohistochemistry. Proteomics analysis in each region of the brain reveals that protein compositions differ among the regions, and these differences in protein expression may be involved in distinct biological actions. Further investigations are needed to elucidate the molecules involved in the biological actions that take place in each region of the brain.

## Experimental Procedures

### Materials

Anti-GFAP, anti-GAT 3, and anti-synaptophysin antibodies were from Sigma-Aldrich (St. Louis, MO). Anti-HSP 70, anti-HSP 90, and anti-α-enolase antibodies were from Santa Cruz Biotechnology Inc. (Santa Cruz, CA). The anti-synaptotagmin antibody was from BD Transduction Laboratories (Mississauga, ON). The anti-Septin 5 antibody was from Abcam (Cambridge, MA). The anti-pyruvate kinase antibody was from Chemicon International, Inc (Temecula, CA). Sequencing grade-modified trypsin was from Promega Corp. (Madison, WI). All other chemicals were commercially available.

### Animals

Sprague-Dawley rats were housed in individual plastic cages (40 × 25 × 25 cm) with wood chip bedding in a room with a 12 h light cycle (12:12 light-dark) maintained at 22°C. Animals had free access to food pellets and tap water. All experiments were accordance with the standards of the Committee for Ethics on Animal Experiments at Yamaguchi University School of Medicine. Rats were randomly assigned from a group, and were anesthetized with Nembutal (40 mg/kg, i.p.). After the anesthetization, rats were transcardially perfused with 0.9% chilled saline, and the brains were removed. In rat brains, cortex, hippocampus, thalamus, and amygdala were dissected, and the cortex were divided into 3 sections, frontal, parietal, and occipital cortexes. The division into six regions: thalamus, hippocampus, frontal cortex, parietal cortex, occipital cortex, and amygdala was carried out according to the atlas of Paxinos and Watson. Samples were homogenized individually in lysis buffer [150 mM Tris (pH 6.8), 12% (w/v) SDS, 36% (v/v) glycerol, and 6% (v/v) 2-mercaptoethanol]. The samples were centrifuged (15,000 × g, 30 min, 4°C), and the supernatants were stored at -20°C.

### In-gel digestion with trypsin

Samples were subjected to SDS-PAGE, and stained lightly with Coomassie Brilliant Blue. In-gel digestion with trypsin was performed as described previously [22,42]. The protein bands in the lanes for samples from the six regions were excised from the gel and divided equally into 24 slices. Each gel slice was diced into small pieces and destained by rinsing in 30% acetonitrile containing 25 mM NH_4_HCO_3_. After the gel pieces were dehydrated in 100% acetonitrile, they were dried naturally at room temperature for 30 min. The proteins in the gel pieces were reduced by incubation with 10 mM dithiothreitol in 25 mM NH_4_HCO_3 _at 56°C for 1 hr, and alkylated with 55 mM iodoacetamide in 25 mM NH_4_HCO_3 _at room temperature for 45 min in the dark. The gel pieces were dehydrated in 50% acetonitrile containing 25 mM NH_4_HCO_3 _twice for 30 min, and then in 100% acetonitrile once for 5 min. After drying for 30 min at room temperature, the gel pieces were rehydrated in TPCK-treated trypsin solution (Trypsin Gold, Mass spectrometry grade, Promega; 10 ng/μl in 25 mM NH_4_HCO_3_) on ice for 30 min. After removing excess solution, digestion was performed overnight at 37°C. The resulting peptides were extracted twice with 50% acetonitrile containing 0.1% trifluoroacetic for 30 min. The extracts were dried in a vacuum concentrator and dissolved in a solution of 0.1% formic acid for mass spectrometric analysis.

### Mass spectrometric analysis

The digested peptides were subjected to LC-MS/MS analysis as described previously [22,24]. Briefly, LC-MS/MS analysis was performed on a Q-Tof Micro (Micromass, Manchester, UK) interfaced with a capillary reverse-phase liquid chromatograph (Micromass CapLC™ system) as described previously [24]. A linear gradient of acetonitrile in 0.1% formic acid was produced and split at a 1:20 ratio. The gradient solution was then injected into a nano LC column (PepMap C18, 75 μm × 150 mm, LC Packings, Amsterdam, Holland) at 100 nl/min. The eluted peptides were sprayed directly into the mass spectrometer. The MS/MS data were acquired by MassLynx software (ver. 4.0; Micromass) using automatic switching between MS and MS/MS modes, and converted to a single text file (containing the observed *m/z *of the precursor peptide, the fragment ion *m/z*, and intensity values) by ProteinLynx software (ver. 2.0; Micromass). The files were analyzed by Mascot MS/MS Ions Search (ver. 3.5; Matrix Science Ltd.) to search and assign the obtained peptides to the NCBI Reference Sequence database (RefSeQ 20060317; 119764 sequences; selected species for the database were *Homo sapiens, Mus musculus*, and *Rattus norvegicus*). Peaks from porcine trypsin, human keratins and bovine serum proteins were removed. We set the parameters as follows: Parent mass error tolerance, ± 0.4 Da; Fragment mass error tolerance, ± 0.2 Da; Enzyme, trypsin; Maximum number of missed cleavages, 1; Fixed post-translational modifications, none; Variable post-translational modifications, oxidation (Met), carbamidomethyl (Cys), propionamide (Cys); Mass values, Monoisotopic. For peptide and protein identification, the search results were processed using a STEM software (STrategic Extractor for Mascot results) as follows [43]. (i) The candidate peptide sequences were screened with the probability-based MOWSE scores that exceeded their thresholds (p < 0.05) and with MS/MS signals for y- or b-ions > 3. (ii) Redundant peptide sequences were removed. (iii) Each peptide sequence was assigned to a protein that gave the maximal number of peptide assignments among the candidates. In this study, visual inspection was applied to individual MS/MS spectra for reliable identification of peptide sequences. Gene names followed the NCBI Gene ID.

### Electrophoresis and immunoblotting

Electrophoresis and immunoblotting were carried out as described previously [21,44,45]. The brain extracts (400 ng) and molecular mass standards were subjected to electrophoresis in 10% (w/v) polyacrylamide gels in the presence of SDS, and transferred to nitrocellulose membranes. The blots were blocked with 5% non-fat dry milk in Tris-buffered saline containing 0.05% (w/v) Tween-20, and incubated with antibody. The blots were then washed, and the antigens were visualized by enhanced chemiluminescent detection reagents.

### Histochemistry and immunohistochemistry

Rat brains were removed, washed with phosphate-buffered solution, and fixed with 4% paraformaldehyde. Then the paraffin-embedded samples were sliced into 4 μm pieces, and stained with hematoxylin and eosin [46,47]. For immunohistochemical observation, the specimens were incubated with 3% H_2_O_2 _in phosphate-buffered saline to quench the endogenous peroxidase activity, and then with blocking solution (Dako, Foster City, CA) to inhibit non-specific binding after deparaffinization. Antigen retrieval was performed in 100% formic acid for 1 min at room temperature, and the samples were incubated with antibodies in 1% bovine serum albumin in phosphate-buffered saline for 1 hr and immunostained by the avidin-biotin peroxidase complex method using a Vectastatin kit (Vector Laboratories, Burlingame, CA). The peroxidase label was visualized by exposing the sections to diaminobenzidine.

### Ingenuity network analysis

The proteins identified in each region were carried out network analysis using Ingenuity Pathways analysis ver.8.6 (IPA, http://www.ingenuity.com). IPA analysis discerns molecular and cellular functions and canonical pathways on the basis of millions findings reported in the literatures, and the software is weekly updated. IPA uses a Fisher's exact test to determine whether the input genes were significantly related to pathways compared to the whole ingenuity knowledge base.

## Abbreviations

LC: liquid chromatography; Q-TOF: quadrupole-time of flight; MS/MS: tandem mass spectrometry; SDS: sodium dodecyl sulfate; PAGE: polyacrylamide gel electrophoresis; GFAP: glial fibrillary acidic protein; GAT 3: GABA transporter 3; HSP: heat shock protein;

## Competing interests

The authors declare that they have no competing interests.

## Authors' contributions

TK, NH, TIzumi, TM, MS, and YM have made substantial contribution to the data acquisition and interpretation in proteomics. HK and TIshihara have participated in the acquisition of the immunohistochemical data. TK, MA, MO, and YM have carried out the experiments of western blotting. YL and SN have contributed to the sample preparations of rat brain. YM have preformed the design of the experiments and have been involved in writing the manuscript. All of authors have read and approved the final manuscript.

## Supplementary Material

Additional file 1 **Table S1**. Identification of proteins in each region of adult rat brain. Rat brains were divided into six regions, and extracted, separated by SDS-PAGE. The samples were subjected to nano-LC-Q-TOF-MS/MS, and analyzed by Mascot search. Mascot scores were subtracted cut off scores, and the localizations and biological functions were searched by NCBI Entrez Gene. Click here for file

Additional file 2 **Table S2**. Lists of ingenuity networks generated by proteins identified in each region of rat brain. Click here for file

Additional file 3 **Figure S1**. Canonical pathways analyzed by proteins identified in each region of rat brain. Click here for file
