# Supplementary material for: Proteomic analysis of proteins expressing in regions of rat brain by a combination of SDS-PAGE with nano-liquid chromatography-quadrupole-time of flight tandem mass spectrometry
Source: Proteome Sci. 2010 Jul 27;8:41. doi: 10.1186/1477-5956-8-41 (PMC2918549; doi:10.1186/1477-5956-8-41)
Supplement: Additional file 1 — Table S1. Identification of proteins in each region of adult rat brain. Rat brains were divided into six regions, and extracted, separated by SDS-PAGE. The samples were subjected to nano-LC-Q-TOF-MS/MS, and analyzed by Mascot search. Mascot scores were subtracted cut off scores, and the localizations and biological functions were searched by NCBI Entrez Gene. [file 1477-5956-8-41-S1.PDF]

**Table. S1 Identification of proteins in each region of adult rat brain.**
Rat brains were divided into six regions, and extracted, separated by SDS-PAGE. The samples were subjected to nano-LC-Q-TOF-MS/MS, and analyzed by Mascot search. Mascot scores were subtracted cut off scores, and the localizations and biological functions were serched by NCBI Entrez Gene

| Mascot score-cut off score= | >100 | >10 | >1 | >0.1 | >0 | 0 |
|---|---|---|---|---|---|---|

| gi(ID) | protein | Protein Mass | Tax. | Thal. | Hipp. | Frot. | Pari. | Occi. | Amyg. | Localizations | Biological functions | Representative Sequences |
|---|---|---|---|---|---|---|---|---|---|---|---|---|
| gil21450321 | Na+/K+ -ATPase alpha 3 subunit | 115894.1 | M | 585.9 | 609.4 | 405.8 | 600.2 | 414.1 | 228.5 | | | DVAGDASESALLK |
| gil109504787 | PREDICTED: similar to tubulin, beta 2 | 49776.0 | R | 224.6 | 394.1 | 304.5 | 473.6 | 476.1 | 403.8 | | | TAVCDIPPR |
| gil62653035 | PREDICTED: similar to Tubulin alpha-2 chain (Alpha-tubulin 2) | 50247.7 | R | 14.9 | 404.5 | 149.4 | 435.0 | 472.5 | 358.7 | | | YMACCLLYR |
| gil32189394 | ATP synthase, H+ transporting, mitochondrial F1 complex, beta subunit precursor | 56524.6 | H | 152.1 | 339.2 | 239.3 | 448.6 | 263.9 | 297.8 | mitochondrion, intracellular | ATP synthesis coupled proton transport | VALVYGGQMNEPPGAR |
| gil21361322 | tubulin, beta 4 | 49553.9 | H | 162.4 | 205.5 | 169.5 | 305.9 | 414.8 | 254.3 | cytoskeleton, protein complex | microtubule-based movement | EVDEQMLSVQSK |
| gil9506497 | clathrin, heavy polypeptide (Hc) | 191476.6 | R | 252.7 | 58.0 | 82.3 | 347.5 | 173.6 | 42.6 | cytoplasm, membrane coat, mitochondrion | intracellular protein transport | VANVELYYK |
| gil112382250 | spectrin, beta, non-erythrocytic 1 isoform 1 | 274460.7 | H | 319.7 | 10.2 | 12.1 | 192.2 | 29.5 | | cytoskeleton, membrane | barbed-end actin filament capping | LQALDTGWNELHK |
| gil40254595 | dihydropyrimidinase-like 2 | 62238.6 | M | 264.6 | 298.5 | 244.2 | 213.8 | 185.3 | 150.1 | axon, cell soma, dendrite, mitochondrion | | SAAEVIAQAR |
| gil42476181 | malate dehydrogenase, mitochondrial | 35660.8 | R | 78.9 | 192.2 | 121.5 | 267.2 | 163.6 | 202.7 | mitochondrion | tricarboxylic acid cycle | IQEAGTEVVK |
| gil4507297 | syntaxin binding protein 1 isoform a | 68692.2 | H | 222.8 | 174.8 | 200.4 | 260.7 | 163.0 | 126.0 | | vesicle-mediated transport | DNALLAQLIQDK |
| gil40538742 | ATP synthase, H+ transporting, mitochondrial F1 complex, alpha subunit, isoform 1. | 59716.6 | O | 43.4 | 260.6 | 107.0 | 229.3 | 216.2 | 173.8 | extracellular region, mitochondrion, intracellular | proton transport | LELAQYR |
| gil40538860 | aconitase 2, mitochondrial | 85380.0 | R | 250.6 | 70.5 | 91.3 | 35.8 | 20.6 | 90.3 | mitochondrion | tricarboxylic acid cycle | ADIANLAEEFK |
| gil82801651 | PREDICTED: similar to heat shock protein 8 | 70926.3 | M | 234.8 | 176.5 | 113.4 | 141.0 | 232.6 | 195.5 | | | VCNPIITK |
| gil41349499 | proteolipid protein 1 isoform 1 | 30057.1 | H | 207.0 | 175.8 | 140.4 | 226.8 | 187.5 | 81.9 | membrane | | GLLECCAR |
| gil82954513 | PREDICTED: similar to heat shock protein 8 | 78981.6 | M | 224.1 | 153.7 | 165.1 | 187.0 | 147.2 | 134.9 | | | MKEIAEAYLGK |
| gil19705431 | albumin | 68674.0 | R | 219.8 | 25.1 | 26.0 | 57.8 | 55.3 | 10.4 | cytoplasm, extracellular region, | body fluid osmoregulation | DNYGELADCCAK |
| gil63029937 | heat shock protein 90kDa alpha (cytosolic), class A member 1 isoform 1 | 98051.5 | H | 215.3 | 74.2 | 139.0 | 89.2 | 28.6 | 114.0 | cytoplasm | protein folding | ELHINLIPNK |
| gil13489067 | N-ethylmaleimide sensitive fusion protein | 82600.0 | R | 148.1 | 209.5 | 81.4 | 167.1 | 40.9 | 173.0 | | | NIDSNPYDTDK |
| gil31560731 | ATPase, H+ transporting, V1 subunit A, isoform 1 | 68282.6 | M | 207.3 | 92.8 | 67.1 | 59.4 | 92.8 | 89.0 | mitochondrion, intracellular | ATP synthesis coupled proton transport, ion transport | EHMGEILYK |
| gil57977323 | cyclic nucleotide phosphodiesterase 1 | 47238.6 | R | 207.0 | 152.5 | 46.4 | 202.0 | 147.5 | 62.5 | membrane | microtubule cytoskeleton organization and biogenesis | HFISGDEPK |
| gil9507159 | synapsin 1 | 73942.6 | R | 206.1 | 119.7 | 109.4 | 155.7 | 161.7 | 76.9 | synapse | neurotransmitter secretion | QGPPQKPPGPAGPIR |
| gil26023949 | enolase 2, gamma | 47110.9 | R | 41.9 | 155.6 | 9.5 | 83.3 | 202.0 | 26.4 | | | IEEELGEEAR |
| gil62657131 | PREDICTED: glial fibrillary acidic protein | 49926.6 | R | 88.2 | 184.0 | | 118.2 | | 2.6 | | | ESASYQEALAR |
| gil28916677 | calcium/calmodulin-dependent protein kinase II alpha isoform 2 | 54080.6 | M | 3.7 | 53.4 | 39.9 | 181.7 | 108.9 | 43.7 | intracellular | protein amino acid phosphorylation, regulation of neurotransmitter secretion | ESSESTNTTIEDEDTK |
| gil94410786 | PREDICTED: similar to Aspartate aminotransferase, mitochondrial precursor (Transaminase A) (Glutamate oxaloacetate transaminase 2) | 53390.1 | O | 6.7 | 22.3 | 114.7 | 168.1 | 57.6 | 73.4 | | | MNLGVGAYR |
| gil82884353 | PREDICTED: similar to Spectrin alpha chain, brain (Spectrin, non-erythroid alpha chain) (Alpha-II s | 285169.7 | O | 164.9 | 11.7 | 82.2 | 120.7 | 47.3 | 32.7 | | | SLQQLAEER |
| gil31981925 | tyrosine 3-monooxygenase/tryptophan 5-monooxygenase activation protein, epsilon polypeptide | 29170.4 | O | 160.3 | 135.4 | 91.3 | 111.5 | 100.3 | 161.0 | | protein targeting | HLIPAANTGESK |

| | | | | | | | | | | | | |
|---|---|---|---|---|---|---|---|---|---|---|---|---|
| gi\|6754206 | hexokinase 1 | 105506.5 | M | 159.7 | 38.9 | 9.3 | 17.5 | 70.0 | 2.8 | cytoplasm, membrane, mitochondrion | glycolysis | LVPDSDVR |
| gi\|6755963 | voltage-dependent anion channel 1 | 30736.6 | M | 4.5 | 159.3 | 48.5 | 17.1 | 20.5 | 38.0 | membrane, mitochondrion | learning,synaptic transmission, transport | YQVDPDACFSAK |
| gi\|25742751 | collapsin response mediator protein 1 | 62156.6 | R | 156.1 | 118.6 | 63.8 | 109.3 | 109.9 | 54.0 | cell soma, dendrite | nervous system development | SIPHITSDR |
| gi\|6978543 | ATPase, Na+/K+ transporting, alpha 1 polypeptide | 112981.9 | R | 31.9 | | 48.0 | 154.6 | 121.7 | 14.6 | membrane | ATP hydrolysis coupled proton transport | LNIPVNQVNPR |
| gi\|30409956 | ATPase, Na+/K+ transporting, alpha 2 polypeptide | 112145.5 | M | 153.5 | 13.1 | 0.0 | 70.8 | 0.2 | 0.0 | membrane | negative regulation of heart contraction, neurotransmitter uptake | DMTSEQLDEILR |
| gi\|61557085 | spectrin beta 2 | 273416.2 | R | 149.7 | 34.6 | 45.4 | 40.4 | | | nucleus, membrane | common-partner SMAD protein phosphorylation | ELEAESYHDIK |
| gi\|31542401 | creatine kinase, brain | 42685.3 | R | 27.8 | 145.2 | 124.3 | 130.3 | 65.0 | 85.5 | cytoplasm, mitochondrion, membrane | brain development, phosphocreatine metabolism | LEQGQPIDDLMPAQK |
| gi\|17921989 | tubulin, alpha 4a | 49892.4 | H | | 44.4 | 36.5 | 137.9 | 59.4 | 6.5 | cytoskeleton, protein complex | microtubule-based movement | DVNAAIAAIK |
| gi\|9506811 | internexin, alpha | 56081.6 | R | 136.9 | 78.9 | 50.7 | 110.1 | 42.1 | | cytoskeleton | cytoskeleton organization, nervous system development | FANLNEQAAR |
| gi\|59853099 | dynamin 1 isoform 1 | 97347.0 | H | 135.9 | 106.4 | 81.0 | 42.9 | 33.5 | 54.1 | membrane, cytoskeleton | receptor mediated endocytosis | NLVDSYMAIVNK |
| gi\|15100179 | malate dehydrogenase 1, NAD (soluble) | 36460.1 | R | 74.0 | 39.3 | 105.2 | 134.0 | 69.0 | 108.5 | cytoplasm | | EVGVYEALK |
| gi\|82894806 | PREDICTED: similar to creatine kinase, brain | 44105.0 | M | | 91.5 | 37.5 | 130.2 | 8.8 | 26.9 | | | LLIEMEQR |
| gi\|51890229 | heat shock 90kDa protein 1, beta | 83264.2 | R | 117.6 | 37.5 | 76.6 | | | 23.5 | cytoplasm, mitochondrion | protein folding | SIYYITGESK |
| gi\|51771467 | PREDICTED: similar to L-lactate dehydrogenase B chain (LDH-B) (LDH heart subunit) (LDH-H) isoform 1 | 36674.2 | O | 116.1 | 45.6 | 48.4 | 103.9 | 4.0 | 20.1 | | | LKDDEVAQLR |
| gi\|82893986 | PREDICTED: similar to Glyceraldehyde-3-phosphate dehydrogenase (GAPDH) | 59781.4 | M | 7.0 | 11.2 | 56.9 | 115.4 | 62.0 | 16.7 | | | GAAQNIIPASTGAAK |
| gi\|62664205 | PREDICTED: similar to grp75 | 73698.8 | R | 114.7 | 3.3 | 9.4 | | 15.5 | 15.6 | | | EQQIVIQSSGGLSK |
| gi\|62657474 | PREDICTED: similar to Actin, cytoplasmic 2 (Gamma-actin) | 62115.1 | R | 102.4 | 69.2 | 12.9 | 102.6 | 114.2 | 76.2 | | | EITALAPSTMK |
| gi\|31982483 | calcium/calmodulin-dependent protein kinase II, beta | 60456.7 | M | | 113.7 | 23.9 | 59.0 | 37.8 | 47.2 | intracellular | calcium ion transport, G1/S transition of mitotic cell cycle | GSLPPAALEPQTTVIHNP VDGIK |
| gi\|110347600 | tubulin, beta-like | 49931.0 | R | | 86.2 | 48.4 | 113.2 | 70.5 | 111.6 | | | INVYYNEAAGNK |
| gi\|18250284 | isocitrate dehydrogenase 3 (NAD+) alpha | 39613.1 | M | | 10.5 | | 113.0 | 45.7 | 19.2 | mitochondrion | tricarboxylic acid cycle | APIQWEER |
| gi\|46485440 | glucose phosphate isomerase | 62787.1 | R | 47.7 | 47.1 | 35.8 | 72.7 | 111.9 | 60.3 | | | HFVALSTNTDK |
| gi\|19705578 | vacuolar H+ATPase B2 | 56514.9 | M | | 15.6 | | 110.6 | 79.0 | 37.9 | cytoplasm, protein complex | ATP synthesis coupled proton transport | AVVQVFEGTSGIDAK |
| gi\|8393823 | neurofilament 3, medium | 95591.5 | R | 108.1 | | | 55.4 | | | axon, cytoskeleton | intermediate filament | EIEAEIHALR |
| gi\|6978549 | ATPase, Na+/K+ transporting, beta 1 polypeptide | 35210.9 | R | 21.5 | 65.7 | 28.8 | 106.7 | 24.2 | 19.2 | membrane | ion transport | YNPNVLPVQCTGK |
| gi\|62643850 | PREDICTED: similar to Eno1 protein | 47502.3 | R | 6.9 | 59.7 | 61.0 | 105.9 | 59.1 | 62.7 | | | SCNCLLLK |
| gi\|6756041 | tyrosine 3-monooxygenase/tryptophan 5-monooxygenase activation protein, zeta polypeptide | 27753.7 | O | 86.3 | 99.3 | 104.7 | 103.5 | 95.1 | 89.3 | mitochondrion, nucleus | protein targeting | YLAEVAAGDDKK |
| gi\|82942194 | PREDICTED: similar to tubulin, alpha 1 | 69491.0 | M | 45.0 | 90.1 | 46.1 | 103.6 | 87.1 | 84.3 | | | DVNAAIATIK |
| gi\|39204499 | neurofilament, light polypeptide | 61471.1 | M | 101.5 | 8.6 | | 101.4 | 36.4 | | cytoskeleton | intermediate filament | TLEIEACR |
| gi\|6981146 | lactate dehydrogenase B | 36589.1 | R | 99.4 | 19.1 | 36.8 | 56.1 | 44.6 | 46.4 | cytoplasm | | MVVDSAYEVIK |
| gi\|62078615 | hypothetical protein LOC299052 | 39467.3 | R | 95.9 | 78.9 | 26.0 | 56.2 | 52.5 | 43.5 | | | ALANSLACQGK |
| gi\|6980970 | glutamate oxaloacetate transaminase 1 | 46299.4 | R | 76.4 | 29.2 | 29.2 | 94.6 | 62.1 | 65.2 | cytoplasm | aspartate catabolism | ITWSNPPAQGAR |
| gi\|6978487 | aldolase A | 39327.3 | R | 1.7 | 94.2 | 7.5 | 0.0 | 8.1 | 55.7 | | fructose metabolism, striated muscle contraction | ELADIAHR |
| gi\|6978489 | aldolase C, fructose-biphosphate | 39259.2 | R | | 91.5 | 36.3 | 54.7 | | 6.7 | mitochondrion | fructose metabolism | ALQASALSAWR |
| gi\|55741544 | ubiquinol-cytochrome c reductase core protein II | 48366.2 | R | 59.2 | 18.8 | | 39.7 | 91.5 | | membrane, mitochondrion | electron transport | AVAFQNPQTR |
| gi\|20302061 | mitochondrial ATP synthase, O subunit | 23382.8 | R | | | | 90.7 | 18.0 | | mitochondrion, intracellular | ATP biosynthesis, hydrogen transport | LVRPPVQVYGIEGR |
| gi\|83030135 | PREDICTED: similar to Triosephosphate isomerase (TIM) (Triose-phosphate isomerase) | 33436.1 | M | 21.3 | 26.7 | 39.1 | 90.5 | 39.9 | | | | IAVAAQNCYK |

| | | | | | | | | | | | | |
|---|---|---|---|---|---|---|---|---|---|---|---|---|
| gil5032139 | synaptotagmin I | 47542.8 | H | 35.4 | 67.1 | 42.9 | 64.9 | 45.6 | 87.4 | membrane, synapse | regulation of exocytosis, synaptic transmission | VPYSELGGK |
| gil56090293 | pyruvate dehydrogenase (lipoamide) beta | 38957.0 | R | | | | 86.8 | 3.8 | 25.6 | mitochondrion | glycolysis | EGIECEVINLR |
| gil19923985 | RAB3C, member RAS oncogene family | 25935.6 | H | 4.7 | 21.0 | | 86.4 | | 0.5 | membrane | small GTPase mediated signal transduction | TITTAYYR |
| gil8393910 | phosphatidylethanolamine binding protein | 20788.4 | R | | 36.1 | | 86.2 | 47.7 | | cell surface | negative regulation of MAPKKK cascade, spermatid development | GNDISSGTVLSEYVGSGP PK |
| gil31377775 | glutamate dehydrogenase 2 | 61395.4 | H | 84.9 | 14.1 | | 33.4 | 0.9 | 33.5 | mitochondrion | electron transport, glutamate metabolism | ALASLMTYK |
| gil33286420 | pyruvate kinase 3 isoform 2 | 58024.9 | H | 22.5 | 81.8 | 47.3 | 49.6 | 56.4 | 43.2 | cytoplasm | glycolysis | GSGTAEVELK |
| gil9507135 | beta-spectrin 3 | 270768.9 | R | | | | 81.5 | | | cytoplasm, cytoskeleton, membrane, nucleus | barbed-end actin filament capping, vesicle-mediated transport | ALAQEDQSAGEVER |
| gil7305305 | N-myc downstream regulated gene 2 | 40763.2 | M | 80.4 | 27.6 | | 48.5 | | 1.2 | | cell differentiation, nervous system development | TASLTSAASIDGSR |
| gil109506410 | PREDICTED: similar to myosin-VIIb | 304679.2 | R | | 79.0 | 58.3 | 60.3 | 60.4 | 66.5 | | | DLTDYLMK |
| gil4506367 | RAB3A, member RAS oncogene family | 24968.1 | H | | 24.4 | | 78.0 | | 58.8 | membrane, cytoplasm | exocytosis, neurotransmitter secretion, small GTPase mediated signal transduction | QLADHLGFEFFEASAK |
| gil117935064 | triosephosphate isomerase 1 | 26903.8 | R | 28.6 | | 50.9 | 19.8 | 77.3 | 15.9 | | fatty acid biosynthesis, gluconeogenesis, | HIFGESDELIGQK |
| gil29788785 | tubulin, beta polypeptide | 49639.0 | H | 27.5 | 49.3 | 75.1 | 62.8 | 48.3 | 52.9 | cytoplasm, cytoskeleton, protein complex | microtubule-based movement, natural killer cell mediated cytotoxicity | ISVYYNEATGGK |
| gil62641274 | PREDICTED: similar to Tu translation elongation factor, mitochondrial | 49391.0 | R | 20.9 | 74.7 | | 12.6 | | | | | AEAGDNLGALVR |
| gil6753428 | creatine kinase, mitochondrial 1, ubiquitous | 46974.2 | M | | 62.3 | 9.0 | 74.7 | 66.8 | 1.6 | mitochondrion | | GTGGVDTAATGSVFDIS NLDR |
| gil27369581 | solute carrier family 25 (mitochondrial carrier, Aralar), member 12 | 74522.7 | M | 25.1 | 48.5 | 5.3 | 72.3 | 74.4 | | membrane, mitochondrion | | GTGSVVGELMYK |
| gil13929094 | guanine deaminase | 50983.8 | R | | 71.7 | | | 19.8 | 3.3 | extracellular region, intracellular | nervous system development, nucleotide and nucleic acid metabolism | DHLLGVSDSGK |
| gil13386272 | citrate synthase-like protein | 52319.9 | M | 28.6 | 0.8 | 35.4 | 41.7 | 71.5 | 53.8 | cytoplasm | tricarboxylic acid cycle | GYSIPECQK |
| gil62651904 | PREDICTED: tumor rejection antigen gp96 (predicted) | 92713.4 | R | 35.4 | 70.9 | 33.0 | | | | | | ELISNASDALDK |
| gil109472265 | PREDICTED: similar to CD207 antigen, langerin | 61380.7 | R | 69.6 | 44.0 | 50.5 | | 37.9 | 53.7 | | | IVSNASCTTNCLAPLAK |
| gil6679651 | enolase 3, beta muscle | 46995.3 | M | 69.0 | 69.1 | | 33.7 | 18.6 | | cytoplasm | | VNQIGSVTESIQACK |
| gil29789026 | neurofilament, heavy polypeptide | 115279.3 | R | 68.9 | | | 1.0 | 4.6 | | axon, cytoskeleton, mitochondrion | intermediate filament cytoskeleton organization and biogenesis, nervous system development | WEMAAQLR |
| gil6981654 | thymus cell antigen 1, theta | 18160.7 | R | | | 13.8 | 66.6 | 10.0 | 32.1 | cell surface, cytoplasm, growth cone, membrane | angiogenesis, cell-cell adhesion, cytoskeleton organization and biogenesis, focal adhesion formation, positive regulation of GTPase activity, positive regulation of peptidyl-tyrosine phosphorylation | DEGDYMCELR |
| gil119874213 | heat shock 70kDa protein 12A | 74848.0 | H | 19.3 | 38.8 | | 66.2 | 21.8 | 4.5 | | | ETAPTSAYSSPAR |
| gil21464101 | tyrosine 3-monooxygenase/tryptophan 5-monooxygenase activation protein, gamma polypeptide | 28284.9 | O | 57.2 | 47.1 | 52.3 | 42.9 | 64.0 | 52.9 | cytoplasm | cytoskeleton organization and biogenesis, negative regulation of protein kinase activity, regulation of neuron differentiation | YLAEVATGEK |
| gil12963799 | ATPase, H+ transporting, V1 subunit D | 28351.4 | M | | | | 63.3 | | | cytoplasm | ATP synthesis coupled proton transport | FTAGDFSTTVIQNVNK |
| gil4507951 | tyrosine 3/tryptophan 5 -monooxygenase activation protein, eta polypeptide | 28201.0 | H | 40.9 | 35.6 | 8.6 | 50.9 | 62.7 | 53.5 | cytoplasm | glucocorticoid receptor signaling pathway, regulation of dendrite morphogenesis, negative regulation of protein kinase activity, regulation of mitosis, regulation of neuron differentiation | NCNDFQYESK |
| gil89030116 | PREDICTED: similar to tubulin, beta 8 isoform 1 | 53635.0 | H | 29.1 | 43.3 | 29.8 | 32.1 | 61.6 | 38.5 | | | FPGQLNADLR |
| gil56605812 | sirtuin (silent mating type information regulation 2 homolog) 2 | 39293.8 | R | 61.3 | 32.5 | | 41.9 | | | cytoplasm, cytoskeleton, nucleus | cell cycle, histone deacetylation, negative regulation of striated muscle development, redox signal response | EHANIDAQSGSQASNPSA TVSPR |
| gil62665891 | PREDICTED: similar to pyruvate kinase (EC 2.7.1.40) isozyme M2 - rat | 57840.1 | R | 17.7 | 25.4 | 27.8 | 61.2 | 24.5 | 14.7 | | | VNLAMNVGK |
| gil19526818 | solute carrier family 25 (mitochondrial carrier, phosphate carrier), member 3 | 39606.5 | M | 9.0 | 28.6 | | 20.4 | 13.1 | 60.5 | membrane, mitochondrion | transport | GSTASQVLQR |

| | | | | | | | | | | | | |
|---|---|---|---|---|---|---|---|---|---|---|---|---|
| gi\|6754012 | guanine nucleotide binding protein, alpha o | 40058.9 | M | 25.2 | 21.8 | 4.6 | 60.5 | 0.6 | 18.9 | membrane | dopamine receptor signaling pathway,locomotory behavior | IIHEDGFSGEDVK |
| gi\|88953559 | PREDICTED: similar to Prostate, ovary, testis expressed protein on chromosome 2 isoform 1 | 121366.7 | O | 53.5 | 47.2 | 26.0 | 59.4 | 59.3 | 55.0 | | | AGFAGDDAPR |
| gi\|4503571 | enolase 1 | 47139.3 | H | 52.9 | 59.0 | | | 41.8 | | cytoplasm, nucleus | glycolysis, regulation of transcription, DNA-dependent, transcription | YNQLLR |
| gi\|39930503 | ATP synthase, H+ transporting, mitochondrial F1 complex, gamma subunit | 32975.1 | R | | | 37.6 | 32.5 | 22.6 | 58.9 | mitochondrion, intracellular | ion transport, oxidative phosphorylation, proton transport | THSDQFLVSFK |
| gi\|13591874 | guanine nucleotide-binding protein, beta-1 subunit | 37363.0 | R | | | | 57.7 | | | membrane | acetylcholine receptor signaling, Ras protein signal transduction, sensory perception of taste | ACADATLSQITNNIDPVGR |
| gi\|4885063 | fructose-bisphosphate aldolase C | 39431.2 | H | 38.8 | 57.5 | | 33.8 | 57.5 | 43.5 | | fructose metabolism | DNAGAATEEFIK |
| gi\|33469051 | tubulin polymerization-promoting protein | 23560.2 | M | | 3.3 | | 56.6 | 7.7 | | | | VDLVDESGYVPGYK |
| gi\|76559899 | glutamine synthetase 1 | 46456.4 | R | 30.6 | 56.6 | 16.8 | 39.0 | 36.1 | | mitochondrion | ammonia assimilation cycle, regulation of neurotransmitter levels | DIVEAHYR |
| gi\|62644670 | PREDICTED: hypothetical protein XP_579688 | 284462.3 | R | 46.3 | 21.2 | 18.6 | 55.5 | | | | | VNEVNQFAAK |
| gi\|73486658 | aspartate aminotransferase 2 precursor | 47487.3 | H | | | | 54.0 | 19.1 | | mitochondrion | aspartate catabolism, biosynthesis | ASAELALGENSEVLK |
| gi\|82801095 | PREDICTED: similar to Fructose-bisphosphate aldolase A (Muscle-type aldolase) | 11133.8 | M | | 53.9 | | 23.4 | | 1.5 | | | FSNEEIAMATVTALR |
| gi\|31982681 | sirtuin 2 (silent mating type information regulation 2, homolog) 2 | 43238.5 | M | 53.7 | 47.8 | | 43.0 | | | cytoplasm, cytoskeleton | cell division, histone deacetylation, mitosis, negative regulation of striated muscle development, redox signal response | FFSCMQSDFSK |
| gi\|8394331 | superoxide dismutase 2 | 24658.6 | R | | | | 53.0 | 36.6 | 31.9 | mitochondrion | regulation of transcription from RNA polymerase II promoter,response to superoxide | AIWNVINWENVSQR |
| gi\|57977273 | phosphofructokinase, platelet | 85665.4 | R | 52.7 | 35.5 | | | | | cytoplasm | | NVLGHMQQGGAPSPFDR |
| gi\|40556608 | heat shock protein 1, beta | 83229.1 | M | 52.7 | | 25.7 | | | 3.5 | mitochondrion | protein folding, response to heat | IDIIPNPQER |
| gi\|88947784 | PREDICTED: similar to Triosephosphate isomerase (TIM) (Triose-phosphate isomerase) isoform 3 | 26982.0 | O | 39.2 | 41.7 | 52.5 | 41.9 | 45.3 | 39.2 | | | IIYGGSVTGATCK |
| gi\|31981722 | heat shock 70kD protein 5 (glucose-regulated protein) | 72378.4 | M | 18.7 | 45.8 | 51.9 | 36.3 | 0.9 | | endoplasmic reticulum | ER overload response, protein folding, response to unfolded protein | NQLTSNPENTVFDAK |
| gi\|41281685 | guanine nucleotide binding protein, alpha activating polypeptide O | 40047.1 | H | | 6.9 | 3.7 | 18.5 | 23.5 | 51.9 | | muscle contraction, nervous system development | IGAADYQPTEQDILR |
| gi\|11693170 | 2-oxoglutarate carrier | 34221.9 | R | | | 9.5 | 51.8 | | 18.8 | membrane, mitochondrion | transport | GIYTGLSAGLLR |
| gi\|6715568 | protein phosphatase 3 (formerly 2B), catalytic subunit, alpha isoform (calcineurin A alpha) | 58650.3 | O | | 34.5 | 32.6 | 17.4 | 51.7 | | protein complex, nucleus | protein amino acid dephosphorylation | GLTPTGMLPSGVLSGGK |
| gi\|34856315 | PREDICTED: similar to ATPase, H+ transporting, V1 subunit B, isoform 1 | 56814.7 | R | 25.8 | 12.1 | | | 51.4 | 6.8 | | | TPVSEDMLGR |
| gi\|13928706 | neural cell adhesion molecule 1 | 94599.2 | R | 5.3 | | 0.5 | 51.0 | 16.2 | | extracellular region, growth cone, membrane | calcium-independent cell-cell adhesion, synaptic transmission | FFLCQVAGDAK |
| gi\|25453412 | glutathione S-transferase, pi 2 | 23424.1 | R | | | | 49.7 | | | | | EAALVDMVNDGVEDLR |
| gi\|62655013 | PREDICTED: hypothetical protein XP_579451 | 60967.3 | R | | 27.8 | | 49.5 | | 13.9 | | | ISSVQSIVPALEIANAHR |
| gi\|21704020 | NADH dehydrogenase (ubiquinone) Fe-S protein 1 | 79697.5 | M | 49.2 | | | 2.0 | | | mitochondrion | | GNDMQVGTYIEK |
| gi\|14149619 | glycoprotein M6A isoform 1 | 31188.4 | H | 18.3 | 3.9 | 32.1 | 34.8 | 48.9 | 19.7 | cell surface, membrane | | SKEEQELHDIHSTR |
| gi\|109505185 | PREDICTED: similar to tubulin, alpha-like 3 | 74401.0 | R | | 48.7 | | | | | | | SFGGGTGSGFTSLLMER |
| gi\|78126167 | solute carrier family 1 (glial high affinity glutamate transporter), member 2 isoform a | 62079.7 | O | 20.1 | 43.6 | 14.6 | 48.2 | 29.5 | 35.3 | dendrite, membrane, synapse | glutamate transport | SELDTIDSQHR |
| gi\|6679939 | glyceraldehyde-3-phosphate dehydrogenase, spermatogenic | 47412.1 | M | 41.6 | 34.9 | 47.0 | 48.2 | 43.2 | 32.8 | flagellum (sensu Eukaryota) | positive regulation of glycolysis, sperm motility | VPTPNVSVVDLTCR |
| gi\|13162302 | dynactin 1 | 141842.0 | R | 48.1 | | | | | | cytoskeleton | microtubule-based process | AEITDAEGLGLK |
| gi\|61098212 | ubiquitin carboxy-terminal hydrolase L1 | 24822.4 | R | 48.0 | 27.8 | 42.4 | 18.5 | 34.0 | 5.6 | axon, cell soma, cytoplasm | adult walking behavior, eating behavior, protein deubiquitination, response to stress, sensory perception of pain, ubiquitin cycle | MQLKPMEINPEMLNK |

| | | | | | | | | | | | | |
|---|---|---|---|---|---|---|---|---|---|---|---|---|
| gil25742568 | dihydropyrimidinase-like 3 | 61928.0 | R | 47.7 | 40.7 | | | | | growth cone, vesicle | axon guidance, neurite development, vesicle-mediated transport | SAADLISQAR |
| gil48255951 | plasma membrane calcium ATPase 2 isoform a | 136789.4 | H | | | | 47.7 | 1.1 | | membrane | | ADVGFAMGIAGTDVAK |
| gil34869154 | PREDICTED: similar to ATPase, H+ transporting, V1 subunit A, isoform 1 | 68221.6 | R | 11.9 | 10.5 | 2.4 | 47.6 | | | | | LSMVQVWPVR |
| gil16418379 | syntaxin 1B2 | 33223.8 | H | 47.5 | | | 11.5 | | | membrane | neurotransmitter transport | QALNEIETR |
| gil51944966 | ATPase, H+/K+ exchanging, alpha polypeptide | 114045.2 | H | 47.5 | | | | 43.2 | | membrane | potassium ion transport | VDNSSLTGESEPQTR |
| gil113199771 | myelin oligodendrocyte glycoprotein | 28252.8 | M | 47.5 | | | 28.4 | 26.4 | | membrane | | ALVGDEAELPCR |
| gil109480990 | PREDICTED: similar to SH3-domain binding protein 1 (3BP-1) | 95625.6 | R | | | | | | 47.3 | | | TPGTGSLAAAVETASGR |
| gil70794816 | hypothetical protein LOC433182 | 47111.2 | M | | 47.1 | 27.1 | 11.5 | 37.2 | 36.4 | | | YITPDQLADLYK |
| gil10092608 | glutathione S-transferase, pi 1 | 23594.1 | M | | | | 47.0 | | | | glutathione metabolism | FEDGDLTLYQSNAILR |
| gil17105366 | synaptic vesicle glycoprotein 2a | 82652.0 | R | 46.8 | | | 12.0 | | | membrane, synapse | neurotransmitter transport | GGLSDGEGPPGGR |
| gil4504067 | aspartate aminotransferase 1 | 46218.5 | H | 46.7 | 8.8 | | 7.0 | 32.0 | 25.4 | cytoplasm | aspartate catabolism, biosynthesis | VGGVQSLGGTGALR |
| gil21735621 | mitochondrial malate dehydrogenase precursor | 35480.7 | H | | | 46.4 | | | | mitochondrion | glycolysis, malate metabolism, tricarboxylic acid cycle | VDFPQDQLTALTGR |
| gil28460695 | eukaryotic translation elongation factor 1 alpha 2 | 50118.1 | R | 45.6 | 26.2 | | 28.8 | 20.5 | 15.2 | | | MDSTEPPYSQK |
| gil31560513 | neurochondrin | 78860.7 | M | 37.6 | 44.7 | | 16.1 | 19.4 | | cellular_component | bone resorption | LLSTSPALQGTPASR |
| gil61556754 | B-cell receptor-associated protein 37 | 33291.9 | R | 44.7 | | | | | 3.9 | membrane, mitochondrion, nucleus | regulation of transcription, DNA-dependent, signal transduction | IVQAEGEAEAAK |
| gil51948476 | ubiquinol-cytochrome c reductase core protein I | 52815.4 | R | 44.7 | 19.6 | 30.6 | 36.6 | 19.2 | | mitochondrion, protein complex | mitochondrial electron transport, ubiquinol to cytochrome c | MVLAAAGGVK |
| gil51766670 | PREDICTED: similar to 60 kDa heat shock protein, mitochondrial precursor (Hsp60) (60 kDa chaperonin | 61016.5 | O | 34.1 | 44.3 | | 37.6 | 42.1 | | | | VGGTSDVEVNEK |
| gil51708092 | PREDICTED: similar to phosphoglycerate kinase 1 | 44522.0 | M | 44.2 | 44.2 | | 8.2 | 43.5 | | | | SLMDEVVK |
| gil4505621 | prostatic binding protein | 21043.7 | H | | 30.1 | | 43.7 | | | | | LYTLVLTDPDAPSR |
| gil28467005 | heat shock protein 1, alpha | 84761.8 | R | 43.7 | | | | 0.8 | | cytoplasm | protein folding, response to unfolded protein | HIYFITGETK |
| gil55926203 | isocitrate dehydrogenase 3 (NAD+) beta | 42326.8 | R | 43.6 | | | 23.3 | | | extracellular region, mitochondrion, | metabolism, tricarboxylic acid cycle | HPFAQAVGR |
| gil20357529 | guanine nucleotide-binding protein, beta-2 subunit | 37307.1 | H | | | 14.2 | 43.2 | 32.8 | 16.4 | | signal transduction | TFVSGACDASIK |
| gil11055998 | guanine nucleotide-binding protein, beta-4 subunit | 37543.0 | H | 25.2 | 29.0 | 43.1 | 42.6 | | 26.2 | | signal transduction | LLVSASQDGK |
| gil15991831 | hexokinase 1 isoform HKI-ta/tb | 102672.2 | H | 42.9 | | | 4.2 | 16.1 | | membrane | glycolysis | FNTSDVSAIEK |
| gil16923964 | contactin 1 | 113423.1 | R | 42.5 | | | 33.2 | 2.1 | 19.2 | extracellular region, membrane, | cell adhesion, DNA methylation, Notch signaling pathway | IVESYQIR |
| gil83030206 | PREDICTED: similar to tubulin, beta 3 | 49969.9 | M | 28.6 | 21.5 | | 42.4 | 30.3 | | | | EVDEQMLAIQSK |
| gil82534351 | microtubule-associated protein tau isoform 1 | 78853.8 | H | 42.1 | | | | | 6.8 | axon, cytoskeleton, cytoplasm, growth cone, membrane | generation of neurons, negative regulation of microtubule depolymerization, positive regulation of axon extension | TPSLPTPPTR |
| gil88957916 | PREDICTED: similar to 14-3-3 protein epsilon (14-3-3E) (Mitochondrial import stimulation factor L s | 29584.7 | O | 7.5 | 34.0 | 5.0 | 41.9 | 9.2 | 35.4 | | | IISSIEQK |
| gil114326546 | phosphoglycerate mutase 1 | 28918.9 | M | 16.9 | 14.6 | | 41.8 | | | | glycolysis, metabolism | FSGWYDADLSPAGHEEA K |
| gil6678551 | vesicle-associated membrane protein 2 | 12682.7 | M | | | | | | 41.6 | membrane, cytoplasm, synapse, synaptosome | calcium ion-dependent exocytosis | ADALQAGASQFETSAAK |
| gil11321585 | guanine nucleotide-binding protein, beta-1 subunit | 37353.0 | H | 25.3 | 21.7 | 21.9 | 41.3 | 14.9 | 22.0 | | signal transduction | LFVSGACDASAK |
| gil5174735 | tubulin, beta, 2 | 49799.0 | H | 30.4 | 41.2 | 26.6 | 35.9 | 34.3 | 26.0 | cytoplasm, cytoskeleton, protein complex | natural killer cell mediated cytotoxicity, protein polymerization | INVYYNEATGGK |
| gil6756039 | tyrosine 3-monooxygenase/tryptophan 5-monooxygenase activation protein, theta polypeptide | 27760.8 | O | | 39.5 | | 41.0 | 12.6 | 36.2 | cellular_component | small GTPase mediated signal transduction | AVTEQGAELSNEER |
| gil9507115 | solute carrier family 1 (glial high affinity glutamate transporter), member 3 | 59644.7 | R | | | | 39.3 | | 41.0 | cell projection, cell soma, dendrite, membrane, synapse | dicarboxylic acid transport, neurotransmitter uptake | IVQVTAADAFLDLIR |

| | | | | | | | | | | | | |
|---|---|---|---|---|---|---|---|---|---|---|---|---|
| gi\|117938759 | guanine nucleotide binding protein, alpha stimulating activity polypeptide 1 isoform c | 97700.5 | O | | 40.6 | 14.8 | 31.5 | 13.9 | 21.6 | cytoplasm, extracellular region, membrane | G-protein coupled receptor protein signaling pathway, pregnancy, protein secretion | LLLLGAGESGK |
| gi\|6978771 | dynamin 2 | 98169.1 | R | 2.7 | 40.6 | | 18.3 | 31.9 | 4.2 | cytoplasm, cytoskeleton, membrane | endocytosis, G2/M transition of mitotic cell cycle, synaptic vesicle transport | GYIGVVNR |
| gi\|13591886 | microtubule-associated protein 1 A | 299348.3 | R | 40.6 | | | | | | cytoplasm, cytoskeleton | negative regulation of microtubule depolymerization, sensory perception of sound | DTDLQQTQATEPR |
| gi\|77917546 | inner membrane protein, mitochondrial | 67135.2 | R | 38.8 | | | | | | mitochondrion | | SLEDALNQTATVTR |
| gi\|62657757 | PREDICTED: similar to pyruvate kinase 3 | 84872.1 | R | 1.3 | 38.7 | 2.5 | 31.3 | 10.0 | | | | IQLINNMLDK |
| gi\|82973377 | PREDICTED: synaptojanin 1 | 187522.9 | M | 7.4 | | | 38.6 | | | | | VSEQTLQSASSK |
| gi\|21361657 | protein disulfide isomerase-associated 3 precursor | 56746.8 | H | 38.6 | | | 6.6 | | | endoplasmic reticulum | protein import into nucleus, protein retention in ER, signal transduction | DGEEAGAYDGPR |
| gi\|78711838 | adaptor-related protein complex 2, beta 1 subunit isoform a | 105654.6 | M | | 8.0 | | | 38.6 | | cytoplasm | intracellular protein transport, protein complex assembly | LHDINAQMVEDQGFLDSLR |
| gi\|62645998 | PREDICTED: similar to N-ethylmaleimide sensitive fusion protein attachment protein beta | 39865.8 | O | | 38.1 | | 1.6 | | 26.3 | | | VAAYAAQLEQYQK |
| gi\|19705465 | ATP synthase, H+ transporting, mitochondrial F0 complex, subunit b, isoform 1 | 28850.5 | R | 23.2 | 37.4 | | | 14.9 | 6.7 | mitochondrion, intracellular | ion transport, proton transport | HVIQSISAQQEK |
| gi\|54020666 | isocitrate dehydrogenase 3, gamma | 42823.2 | R | | | | 37.4 | | | mitochondrion | negative regulation of growth, tricarboxylic acid cycle | LGDGLFLQCCR |
| gi\|89035672 | PREDICTED: similar to Phosphoglycerate mutase 1 (Phosphoglycerate mutase isozyme B) (PGAM-B) (BPG-d | 28831.8 | O | | | | 37.3 | | | | | ALPFWNEEIVPQIK |
| gi\|8393502 | glutathione S-transferase, mu 1 | 25897.1 | R | | 37.2 | 13.8 | | 6.3 | 11.6 | extracellular region | sensory perception, sensory perception of smell | LLLEYTDSSYEEK |
| gi\|8394027 | alpha isoform of regulatory subunit A, protein phosphatase 2 | 65280.9 | M | | | | 37.1 | | | cytoplasm | | IGPILDNSTLQSEVKPILEK |
| gi\|67078422 | thioredoxin domain containing 1 | 31414.7 | R | 36.7 | | | | | | | electron transport | VDVTEQTGLSGR |
| gi\|62638908 | PREDICTED: similar to tweety 1 | 49001.1 | R | | | | | | 36.6 | | | TELTTLEEVLSER |
| gi\|34866853 | PREDICTED: similar to cytochrome c-1 | 35412.0 | R | | | | 35.8 | | | | | GLLSSLDHTSIR |
| gi\|34538601 | cytochrome c oxidase subunit II | 25959.0 | M | | | | | 35.7 | 22.6 | | | ILYMMDEINNPVLTVK |
| gi\|82917120 | PREDICTED: similar to cytoplasmic beta-actin isoform 2 | 26448.2 | M | | | 35.5 | | | | | | QEYDESGPSIVHR |
| gi\|109465389 | PREDICTED: similar to Moloney leukemia virus 10 | 113629.0 | R | | | | 35.0 | | 5.3 | | | LLLNNDNLLR |
| gi\|82942469 | PREDICTED: similar to tubulin, alpha 1 | 54777.3 | M | | 13.5 | 18.7 | 34.9 | 19.0 | 13.5 | | | QLFHPEQLITGK |
| gi\|32189355 | solute carrier family 25, member 4 | 32968.2 | R | | 21.8 | 18.0 | 21.0 | 34.6 | 3.3 | membrane, mitochondrion | ADP transport, ATP transport, glutamate uptake during transmission of nerve impulse, mitochondrial genome maintenance, transport | AAYFGVYDTAK |
| gi\|9507167 | synaptogyrin 1 | 25652.3 | R | 21.0 | 8.7 | 4.9 | 34.5 | 9.9 | | membrane, synapse | protein targeting | DNPLNEGTDAAR |
| gi\|62234487 | plasma membrane calcium ATPase 1 | 134661.9 | M | 34.4 | 7.7 | | 19.4 | 15.2 | | membrane | | QVVAVTGDGTNDGPALK |
| gi\|27702072 | PREDICTED: similar to NADH-ubiquinone oxidoreductase 30 kDa subunit, mitochondrial precursor (Compl | 30207.6 | O | | | 34.3 | | | 3.8 | | | SLADLTAVDVPTR |
| gi\|31377489 | dynein, cytoplasmic, heavy chain 1 | 531915.8 | R | 34.1 | | | 7.0 | | | cytoskeleton | microtubule-based movement, proteolysis | VQGLTVEQAEAVAR |
| gi\|4505685 | pyruvate dehydrogenase (lipoamide) alpha 1 | 43267.6 | H | | 31.7 | 11.1 | 10.5 | | 34.0 | mitochondrion | acetyl-CoA metabolism, glycolysis, metabolism | LEEGPPVTTVLTR |
| gi\|53692187 | actin-related protein 2 isoform a | 45347.6 | H | | 33.8 | | | | 16.6 | cytoskeleton | | LCYVGYNIEQEQK |
| gi\|82887201 | PREDICTED: similar to Tubulin alpha-2 chain (Alpha-tubulin 2) | 28075.8 | M | | | | | | 33.7 | | | MVDNEAIYDICR |
| gi\|11321583 | succinate-CoA ligase, ADP-forming, beta subunit | 50285.2 | H | 33.6 | 13.6 | | | 15.3 | | mitochondrion | succinyl-CoA pathway | ICNQVLVCER |
| gi\|45504359 | vacuolar H+ ATPase E1 | 26140.8 | M | 11.7 | 4.9 | 7.0 | 33.6 | | 24.5 | cytoplasm, mitochondrion, protein complex | ATP synthesis coupled proton transport, | LDLIAQQMMPEVR |
| gi\|58743306 | alpha-tubulin isotype H2-alpha | 49987.7 | H | | | | 33.2 | 1.1 | 21.8 | cytoskeleton, protein complex | microtubule-based movement, protein polymerization | LIGQIVSSITASLR |

| | | | | | | | | | | | | |
|---|---|---|---|---|---|---|---|---|---|---|---|---|
| gi\|6679299 | prohibitin | 29801.9 | M | 33.1 | | | | | | membrane, mitochondrion | DNA replication | IYTSIGEDYDER |
| gi\|4502855 | sarcomeric mitochondrial creatine kinase precursor | 47490.4 | H | | 20.4 | 0.0 | 33.1 | 25.8 | 12.9 | mitochondrion | generation of precursor metabolites and energy, muscle contraction | LGYILTCPSNLGTGLR |
| gi\|82884325 | PREDICTED: similar to Spectrin alpha chain, brain (Spectrin, non-erythroid alpha chain) (Alpha-II s | 60279.1 | O | | | 16.8 | 32.7 | 9.7 | | | | DLSSVQTLLTK |
| gi\|16757994 | pyruvate kinase, muscle | 57780.9 | R | 32.5 | 10.4 | 5.8 | 6.2 | 7.1 | | cytoplasm, mitochondrion | glycolysis | EAEAAVFHR |
| gi\|18543177 | citrate synthase | 51833.5 | R | 31.7 | 32.5 | | 12.9 | 4.6 | 13.5 | mitochondrion | | EGSSIGAIDSK |
| gi\|61097906 | actinin, alpha 1 | 103003.6 | M | | | | 32.3 | | | cytoskeleton, cytoplasm, membrane | cortical cytoskeleton organization and biogenesis | GISQEQMNEFR |
| gi\|23346461 | NADH dehydrogenase (ubiquinone) Fe-S protein 2 | 52591.7 | M | | 31.8 | | | | | mitochondrion | electron transport | APGFAHLAGLDK |
| gi\|4506931 | SH3-domain GRB2-like 2 | 39937.1 | H | | 28.5 | | 31.6 | 2.4 | 30.8 | cytoplasm | central nervous system development | QAVQILQQVTVR |
| gi\|9506445 | carbonic anhydrase 2 | 29095.6 | R | 31.5 | | | | 2.2 | | cytoplasm | carbon dioxide transport, morphogenesis of an epithelium, osteoclast differentiation | IGPASQGLQK |
| gi\|13786202 | voltage-dependent anion channel 2 | 31725.6 | R | 1.6 | | 29.3 | 31.5 | 30.3 | 26.0 | mitochondrion | anion transport | LTFDTTFSPNTGK |
| gi\|109487858 | PREDICTED: similar to Synaptogyrin 3 | 24497.4 | R | 31.1 | | | | | | | | TAPGPGTAQAGDAAR |
| gi\|109475599 | PREDICTED: similar to 14-3-3 protein sigma (Stratifin) | 36725.0 | R | | 30.7 | 9.9 | 29.6 | 5.8 | 10.0 | | | NLLSVAYK |
| gi\|6679186 | claudin 11 | 22099.3 | M | 30.0 | 17.5 | | 30.6 | | | membrane | axon ensheathment, calcium-independent cell-cell adhesion, cell adhesion, spermatogenesis | FYYSSGSSSPTHAK |
| gi\|9506411 | ATP synthase, H+ transporting, mitochondrial F0 complex, subunit d | 18751.6 | R | | | | 30.5 | | 7.8 | mitochondrion, intracellular | ion transport, proton transport | NCAQFVTGSQAR |
| gi\|7657116 | glyceraldehyde-3-phosphate dehydrogenase, spermatogenic | 44472.8 | H | | 30.4 | | | 20.4 | | | positive regulation of glycolysis, sperm motility | VPTPDVSVVDLTCR |
| gi\|14043072 | heterogeneous nuclear ribonucleoprotein A2/B1 isoform B1 | 37406.7 | H | | | 7.9 | 29.6 | 11.9 | 30.1 | nucleus | nuclear mRNA splicing | IDTIEIITDR |
| gi\|34419635 | heat shock 70kDa protein 6 (HSP70B') | 70984.2 | H | 12.0 | 13.8 | 11.9 | 13.0 | 14.4 | 29.8 | | protein folding | TTPSYVAFTDTER |
| gi\|45598372 | brain abundant, membrane attached signal protein 1 | 22073.6 | M | 0.0 | 1.0 | 23.5 | 29.1 | 4.8 | | | | ESEPQAAADATEVK |
| gi\|16758892 | calbindin 2 | 31384.5 | R | 28.2 | | | | | | membrane | | EMNIQQLTTYR |
| gi\|41393608 | reticulon 3 isoform b | 110583.5 | H | | | | 27.7 | | 2.7 | endoplasmic reticulum, extracellular region, membrane | | TQIDHYVGIAR |
| gi\|23510338 | ubiquitin-activating enzyme E1 | 117774.3 | H | 9.3 | 27.5 | | | | | | DNA replication, ubiquitin cycle | LAGTQPLEVLEAVQR |
| gi\|40445397 | beta-glo | 16027.2 | R | | | | | | 27.4 | | | VVAGVASALAHK |
| gi\|13591955 | guanine nucleotide binding protein, alpha inhibiting 2 | 40473.1 | R | | 27.4 | | | | | | acetylcholine receptor signaling, adenylate cyclase inhibiting pathway, | LFDSICNNK |
| gi\|29788768 | tubulin, beta polypeptide paralog | 49920.9 | H | 27.3 | 8.4 | | | 20.7 | | cytoplasm, cytoskeleton, protein complex | microtubule-based movement | INVYYNEATGNK |
| gi\|82884323 | PREDICTED: similar to Spectrin alpha chain, brain (Spectrin, non-erythroid alpha chain) (Alpha-II s | 147141.8 | O | 12.7 | 8.0 | 27.2 | 19.7 | 5.1 | | | | EELYQNLTR |
| gi\|109507853 | PREDICTED: similar to vacuolar protein sorting 35 | 91668.8 | R | 27.1 | | | | | | | | EDGPGIPAEIK |
| gi\|45827776 | reticulon 1 isoform C | 23560.6 | H | | | | 26.8 | | | endoplasmic reticulum, membrane | neuron differentiation | SQAIDLLYWR |
| gi\|82884343 | PREDICTED: similar to Spectrin alpha chain, brain (Spectrin, non-erythroid alpha chain) (Alpha-II s | 235125.0 | O | 26.4 | 4.2 | | | | | | | DLTNVQNLQK |
| gi\|15147224 | sideroflexin 1 | 35626.3 | M | | | | | | 26.3 | membrane, mitochondrion | cation transport, erythrocyte differentiation | NILLTNEQLENAR |
| gi\|11560135 | brain abundant, membrane attached signal protein 1 | 21777.4 | R | | | | 20.3 | | 25.8 | membrane, nucleus | regulation of transcription | AGEASAESTGAADGAPQEEGEAK |
| gi\|4758788 | NADH dehydrogenase (ubiquinone) Fe-S protein 3, 30kDa (NADH-coenzyme Q reductase) | 30222.7 | H | | | | 25.7 | | | membrane, mitochondrion | mitochondrial electron transport, NADH to ubiquinone | VVAEPVELAQEFR |
| gi\|8567390 | pyruvate kinase liver and red blood cell | 62269.6 | M | | 25.5 | | | | | | | GDLGIEIPAEK |
| gi\|9257192 | adducin 2 isoform a | 80803.4 | H | | | | 25.3 | | | cytoskeleton, membrane | | VNVADEVQR |

| | | | | | | | | | | | | |
|---|---|---|---|---|---|---|---|---|---|---|---|---|
| gil4503875 | glutamate decarboxylase 2 | 65368.5 | H | 25.1 | | | | | | membrane | glutamate decarboxylation to succinate | HYDLSYDTGDK |
| gil94380294 | PREDICTED: similar to Carcinoembryonic antigen-related cell adhesion molecule 1 precursor (Biliary glycoprotein 1) (BGP-1) (Murine hepatitis virus receptor) (MHV-R) (Biliary glycoprotein D) | 108834.8 | M | 25.0 | | | | | | | | EIDGGLETLR |
| gil62654759 | PREDICTED: phosphoglycerate kinase 2 (predicted) | 44981.3 | R | 3.7 | 24.7 | | 11.7 | 24.9 | 16.0 | | | VDFNVPMK |
| gil4507191 | spectrin, alpha, non-erythrocytic 1 (alpha-fodrin) | 284107.1 | H | 24.8 | 0.8 | | | | | cytoskeleton, membrane | barbed-end actin filament capping | LSDDNTIGKEEIQQR |
| gil82999838 | PREDICTED: similar to zinc finger protein 161 | 33185.2 | M | | | | 24.5 | | | | | LTLSALLDGK |
| gil4506713 | ubiquitin and ribosomal protein S27a precursor | 17953.5 | H | | | | 11.1 | | 24.5 | cytoplasmic small ribosomal subunit | protein biosynthesis, protein modification | TITLEVEPSDTIENVK |
| gil62642907 | PREDICTED: similar to Phosphoglycerate kinase 1 | 43148.3 | R | | | | 24.1 | 20.6 | | | | ALESPERPFLAILGGAK |
| gil5031857 | lactate dehydrogenase A | 36665.4 | H | | | | 24.0 | | 5.1 | cytoplasm, cytosol | tricarboxylic acid | SADTLWGIQK |
| gil62662610 | PREDICTED: similar to Glyceraldehyde-3-phosphate dehydrogenase (GAPDH) | 31224.5 | R | | 24.0 | | | | | | | VVDLMSYMASK |
| gil6679599 | RAB7, member RAS oncogene family | 23543.9 | M | | | | 23.8 | | | Golgi apparatus, cytoplasm, membrane | small GTPase mediated signal transduction, transport | EAINVEQAFQTIAR |
| gil5031573 | ARP3 actin-related protein 3 homolog | 47341.0 | H | 23.6 | | | | | | cytoskeleton | cell motility | LPACVVDCGTGYTK |
| gil11125772 | myristoylated alanine-rich protein kinase C substrate | 31525.8 | H | 19.1 | | | 23.6 | 7.3 | 6.6 | cytoskeleton, membrane | | GEAAAERPGEAAVASSPSK |
| gil12711692 | dihydropyrimidinase-like 5 | 61501.2 | R | | 23.4 | | | | | cell soma, cytoplasm, dendrite, protein complex | axon guidance, nervous system development | TPYLGDVAVVVNPGK |
| gil109506701 | PREDICTED: similar to Gamma-soluble NSF attachment protein (SNAP-gamma) (N-ethylmaleimide-sensitive | 144886.5 | O | | | | 23.2 | | | | | LPEAVQLIEK |
| gil14165437 | heterogeneous nuclear ribonucleoprotein K isoform a | 50996.4 | H | 23.0 | | | | 13.0 | | nucleus, ribonucleoprotein complex | DNA-dependent, transcription | TDYNASVSVPDSSGPER |
| gil6671664 | calnexin | 67235.7 | M | 23.0 | | | | | | endoplasmic reticulum, membrane | protein folding | IADPDAVKPDDWDEDAPSK |
| gil109510373 | PREDICTED: similar to NF-kappaB repressing factor | 143912.0 | O | | 14.0 | | 22.8 | | | | | VNIIPIIAK |
| gil62639687 | PREDICTED: similar to Adapter-related protein complex 2 alpha 1 subunit (Alpha-adaptin A) (Adaptor | 107597.9 | O | 7.5 | | | 22.8 | | | | | YLETADYAIR |
| gil13591900 | 4-aminobutyrate aminotransferase | 56418.5 | R | 4.9 | | | | 22.4 | | mitochondrion, protein complex | behavior, gamma-aminobutyric acid catabolism | ESLMSVAPK |
| gil34859236 | PREDICTED: similar to Proteasome subunit beta type 3 (Proteasome theta chain) (Proteasome chain 13) | 23030.5 | O | | | | 22.4 | | | | | FGPYYTEPVIAGLDPK |
| gil33563256 | guanine nucleotide binding protein, alpha inhibiting 3 | 40512.2 | M | | | | 20.1 | 22.3 | 10.4 | cytoplasm, Golgi apparatus, membrane | G-protein coupled receptor protein signaling pathway, regulation of heart contraction | TTGIVETHFTFK |
| gil6753966 | glycerol-3-phosphate dehydrogenase 1 (soluble) | 37548.4 | M | 22.3 | | | | | | cytoplasm, protein complex | glycerol-3-phosphate metabolism | FCETTIGCK |
| gil5032007 | purine-rich element binding protein A | 34889.4 | H | | 2.3 | 2.1 | | 22.1 | 0.9 | nucleus | G1/S transition checkpoint, DNA-dependent, transcription | FFFDVGSNK |
| gil6754658 | Golli-mbp isoform 1 | 27151.4 | M | 21.8 | | | 15.2 | | | | myelination | TTHYGSLPQK |
| gil62653460 | PREDICTED: similar to L-lactate dehydrogenase A chain (LDH-A) (LDH muscle subunit) (LDH-M) | 36489.3 | O | 0.5 | | 8.3 | 12.4 | | 21.8 | | | VTLTPDEEAR |
| gil10946936 | adenylate kinase 1 | 23101.8 | M | | 21.7 | | 15.2 | | | cytoplasm, mitochondrion, membrane | ATP metabolism, cell cycle arrest | IIFVVGGPGSGK |

| | | | | | | | | | | | | |
|---|---|---|---|---|---|---|---|---|---|---|---|---|
| gil14210536 | tubulin, beta 6 | 49825.0 | H | | 6.1 | | 21.6 | | | cytoplasm, cytoskeleton, protein complex | microtubule-based movement, protein polymerization | GHYTEGAELVDAVLDVVR |
| gil62650366 | PREDICTED: similar to glyceraldehyde-3-phosphate dehydrogenase, spermatogenic | 76910.9 | R | 3.7 | | 5.9 | 21.2 | 14.7 | 15.0 | | | VVDLMAYMASK |
| gil62665162 | PREDICTED: ATPase, H+ transporting, V0 subunit D isoform 1 (predicted) | 51009.9 | R | 21.2 | 12.8 | 16.7 | 5.9 | | 4.7 | | | LLFEGAGSNPGDK |
| gil82996621 | PREDICTED: similar to Triosephosphate isomerase (TIM) (Triose-phosphate isomerase) | 23903.1 | M | | | | 21.1 | | | | | VVLAYELVWAIGTGK |
| gil6752952 | actin, gamma 2, smooth muscle, enteric | 42852.3 | M | | 7.1 | 2.9 | 21.1 | 2.7 | | cytoskeleton | cytoskeleton organization and biogenesis | YPIEHGIITNWDDMEK |
| gil47933379 | N-ethylmaleimide-sensitive factor attachment protein, alpha | 33211.3 | H | 21.1 | | | | | 3.0 | endoplasmic reticulum, Golgi apparatus | ER to Golgi vesicle-mediated transport | TIQGDEEDLR |
| gil69885032 | myelin basic protein isoform 1 | 21489.0 | M | 20.8 | | | | | | | myelination | GAYDAQGTLSK |
| gil53850628 | NADH dehydrogenase (ubiquinone) Fe-S protein 1, 75kDa | 79361.6 | R | 20.6 | 1.9 | | | | | mitochondrion | NADH oxidation | VAGMLQSFEGK |
| gil21328448 | tyrosine 3-monooxygenase/tryptophan 5-monooxygenase activation protein, beta polypeptide | 28064.8 | O | 18.3 | | | 20.5 | 16.5 | 16.7 | cytoplasm | | YLSEVASGDNK |
| gil6980972 | glutamate oxaloacetate transaminase 2 | 47284.1 | R | | 20.1 | 16.2 | | | | cell surface, extracellular region, mitochondrion | aspartate catabolism, fatty acid transport | EYLPIGGLADFCK |
| gil4503971 | GDP dissociation inhibitor 1 | 50550.1 | H | 20.0 | | 2.2 | | 12.0 | | cytoplasm | protein transport, regulation of GTPase activity | TFEGVDPQTTSMR |
| gil5032009 | glycogen phosphorylase | 97030.6 | H | | | | | 19.9 | | | glycogen metabolism | YEFGIFNQK |
| gil31560560 | laminin receptor 1 (ribosomal protein SA) | 32893.5 | M | | | | 19.9 | | | intracellular, ribosome | protein biosynthesis | AIVAIENPADVSVISSR |
| gil31542559 | dihydrolipoamide S-acetyltransferase (E2 component of pyruvate dehydrogenase complex) | 67902.7 | O | | | | 12.3 | 19.9 | | mitochondrion, cytoplasm | acetyl-CoA biosynthesis from pyruvate | VVDGAVGAQWLAEFK |
| gil62661722 | PREDICTED: similar to succinate-Coenzyme A ligase, ADP-forming, beta subunit | 50274.2 | R | 19.5 | 2.5 | | 1.1 | | | | | ILACDDLDEAAK |
| gil31982030 | Rho GDP dissociation inhibitor (GDI) alpha | 23392.8 | M | | 8.6 | | 19.5 | 3.4 | 2.2 | membrane | Rho protein signal transduction | VAVSADPNVPNVIVTR |
| gil62642537 | PREDICTED: similar to mKIAA0417 protein | 74783.5 | R | 16.2 | | | 19.5 | | | | | LDLTGTSGAAVPAR |
| gil8393742 | myelin-associated glycoprotein | 69308.9 | R | 19.3 | | | | | | extracellular region, membrane | cell adhesion | SNPEPSVAFELPSR |
| gil71534276 | guanosine diphosphate dissociation inhibitor 1 | 50504.2 | R | 3.8 | 5.2 | 0.4 | | | 19.3 | membrane | small GTPase mediated signal transduction | KQNDVFGEADQ |
| gil109464792 | PREDICTED: similar to PDZ domain containing 6 | 186746.7 | R | 18.0 | | | 19.1 | 11.0 | | | | YFPTQALNFAFK |
| gil62653546 | PREDICTED: similar to glyceraldehyde-3-phosphate dehydrogenase | 35760.1 | R | | 19.0 | 1.2 | | | 14.3 | | | LVNNGKPITIFQER |
| gil6679066 | 4-nitrophenylphosphatase domain and non-neuronal SNAP25-like protein homolog 1 | 33342.0 | M | | | | | | 18.8 | mitochondrion | biological_process | AGPNIYELR |
| gil62945278 | hypothetical protein LOC360975 | 116221.4 | R | | | 18.8 | | | | | | VIPEDGPAAQNPDK |
| gil31543974 | tyrosine 3-monooxygenase/tryptophan 5-monooxygenase activation protein, beta polypeptide | 28068.9 | O | 18.7 | 13.5 | | 7.8 | | | cellular_component | protein targeting | AVTEQGHELSNEER |
| gil41399285 | chaperonin | 61016.4 | H | | 18.6 | | | | | cytoplasm, mitochondrion | protein import into mitochondrial matrix, regulation of apoptosis | LVQDVANNTNEEAGDGTTTATVLAR |
| gil4759080 | succinate dehydrogenase complex, subunit A, flavoprotein precursor | 72643.3 | H | 18.6 | | | | | | mitochondrion | tricarboxylic acid cycle | GEGGILINSQGER |
| gil11230802 | actinin alpha 4 | 104911.4 | M | | | 0.6 | | 18.5 | | cytoskeleton, cytoplasm, pseudopodium | actin filament bundle formation, positive regulation of pinocytosis | LASDLLEWIR |
| gil30795227 | histidyl-tRNA synthetase 2 | 23409.0 | H | 18.4 | | | | | | cytoplasm | D-amino acid catabolism | SASSGAEGDVSSEREP |
| gil77404242 | synapsin 2 isoform 1 | 63416.8 | R | | 18.4 | | | | 15.8 | synapse | regulation of neurotransmitter secretion, synaptic transmission | TPALSPQRPLTTQQPQSGTLK |
| gil34858950 | PREDICTED: brain glycogen phosphorylase | 96676.5 | R | 18.1 | | | | 13.5 | 3.7 | | | VEDVEALDQK |
| gil82894424 | PREDICTED: similar to heat shock protein 1 (chaperonin) | 67369.3 | M | | | | | 18.0 | | | | LSDGVAVLK |
| gil89060515 | PREDICTED: similar to Phosphoglycerate kinase 1 | 10241.4 | H | | 17.9 | | | | | | | ALESPERPFLAILGGAK |
| gil16758840 | crystallin, mu | 33533.1 | R | | | 17.8 | | | | | visual perception | FASSVQGDVR |
| gil34873499 | PREDICTED: aminopeptidase puromycin sensitive | 103278.8 | R | 17.8 | | | | 4.8 | | | | YTTPAGEVR |

| | | | | | | | | | | | | | |
|---|---|---|---|---|---|---|---|---|---|---|---|---|---|
| gil83700235 | eukaryotic translation initiation factor 4A isoform 2 | 46372.8 | H | | 3.8 | | 17.6 | | | cytoplasm | protein biosynthesis, regulation of translational initiation | VLITTDLLAR |
| gil13994177 | synaptosomal-associated protein, 91kDa homolog | 93460.3 | R | | | | 17.5 | | | membrane | | ATNSSWVVVFK |
| gil19913426 | ATPase, H+ transporting, lysosomal 56/58kDa, V1 subunit B1 | 56796.8 | H | | 17.3 | 0.9 | | 4.4 | 5.3 | cytoplasm, membrane, intracellular | regulation of pH, sensory perception of sound | QIYPPINVLPSLSR |
| gil15042957 | RAB3D, member RAS oncogene family | 24400.9 | M | | | | 2.3 | | 17.2 | intracellular, membrane, cytoplasm | exocytosis, small GTPase mediated signal transduction | LLLIGNSSVGK |
| gil63530505 | predicted gene, EG433923 | 33050.3 | O | 16.9 | 11.5 | 12.1 | 1.2 | | 12.0 | | | LLLQVQHASK |
| gil121583768 | RAB5B, member RAS oncogene family | 23544.0 | R | | | | 16.8 | | | cytoplasm, | regulation of endocytosis | TAMNVNDLFLAIAK |
| gil62664637 | PREDICTED: similar to solute carrier family 25, member 5 | 33155.4 | R | | 0.1 | | 16.7 | | | | | GAWSNVLR |
| gil89024803 | PREDICTED: similar to heat shock protein 8 | 72696.0 | H | | | 13.2 | 6.2 | 16.5 | 4.4 | | | LLQDFFNGK |
| gil6981182 | microtubule-associated protein 2 | 198445.6 | R | 3.5 | | | 16.1 | | | cytoplasm, cytoskeleton, dendrite | microtubule bundle formation | FAAPVQPEEER |
| gil12025532 | ATPase, H+ transporting, lysosomal V0 subunit a isoform 1 | 96231.1 | M | 8.2 | | 0.7 | 15.8 | | | cytoplasm, membrane, nucleus | brain development, heart development, ion transport, myeloid cell differentiation | ASLYPCPETPQER |
| gil61889115 | phosphoserine aminotransferase 1 | 40600.8 | R | 15.7 | | | | | | | L-serine biosynthesis | QVVNFGPGPAK |
| gil40254781 | GDP dissociation inhibitor 2 | 50504.7 | R | 4.0 | 15.7 | | 2.9 | | | cytoplasm, Golgi apparatus | small GTPase mediated signal transduction, vesicle-mediated transport | MLLFTEVTR |
| gil32455264 | peroxiredoxin 1 | 22096.3 | H | | | | 15.6 | | | | cell proliferation, skeletal development | QITVNDLPVGR |
| gil13591908 | adaptor protein complex AP-2, alpha 2 subunit | 103979.1 | R | 15.3 | | | | | | cytoplasm, Golgi apparatus | endocytosis, intracellular protein transport, vesicle-mediated transport | YGGTFQNVSVK |
| gil89034708 | PREDICTED: hypothetical protein XP_947819 | 21260.3 | H | 15.2 | | | 9.9 | | | | | QNYHQDSEAAINR |
| gil54607098 | succinate dehydrogenase Fp subunit | 72539.2 | M | | | 13.4 | | 15.1 | | mitochondrion | electron transport, transport, tricarboxylic acid cycle | TLNEADCATVPPAIR |
| gil21311845 | solute carrier family 25 (mitochondrial carrier, glutamate), member 22 | 34648.2 | M | | | | 15.1 | | | membrane, mitochondrion | glutamate transport, transport | LAANDFFR |
| gil61657921 | kinesin family member 5B | 109483.7 | M | 15.0 | | | | | | cytoplasm, cytoskeleton | microtubule-based process, mitochondrial transport | GGGSFVQNNQPVGLR |
| gil109465638 | PREDICTED: similar to Rap1 GTPase-GDP dissociation stimulator 1 (SMG P21 stimulatory GDP/GTP exchange protein) (SMG GDS protein) (Exchange factor smgGDS) | 87543.0 | R | | | | 15.0 | | | | | SVAQQASLTEQR |
| gil11560087 | liver glycogen phosphorylase | 97421.0 | R | 13.9 | 14.8 | 0.2 | | | | | | VLYPNDNFFEGK |
| gil89036204 | PREDICTED: similar to Ras-related protein Rab-13 | 24807.6 | H | | 12.3 | | | 12.5 | 14.7 | | | LLLIGDSGVGK |
| gil13399308 | related RAS viral (r-ras) oncogene homolog 2 | 23439.8 | M | 14.6 | | | | | | intracellular, membrane | Ras protein signal transduction | QVTQEEGQQLAR |
| gil66346706 | neuronal growth regulator 1 | 38694.3 | H | | | | 14.1 | | | membrane | cell adhesion | VVVNFAPTIQEIK |
| gil88952640 | PREDICTED: similar to ubiquitin and ribosomal protein S27a precursor | 17955.4 | H | | | | | | 14.0 | | | ESTLHLVLR |
| gil109504977 | PREDICTED: similar to Histone H2B 291B | 36920.9 | R | | | | 13.8 | | | | | VFLENVIR |
| gil6678195 | synaptophysin | 33332.1 | M | 10.9 | 12.1 | 13.8 | 3.5 | 12.7 | 11.0 | membrane, synapse, synaptosome | endocytosis, synaptic transmission | MATDPENIIK |
| gil68303561 | proteasome (prosome, macropain) subunit, alpha type, 8 isoform 1 | 28512.1 | H | | | | | 13.7 | | cytoplasm, protein complex | ubiquitin-dependent protein catabolism | ALLEVVQSGGK |
| gil31711992 | dihydrolipoamide S-acetyltransferase (E2 component of pyruvate dehydrogenase complex) | 68953.1 | O | 1.8 | 13.6 | 4.3 | | | 10.9 | mitochondrion, cytoplasm | acetyl-CoA biosynthesis | ILVPEGTR |
| gil48762932 | chaperonin containing TCP1, subunit 8 (theta) | 59582.5 | H | | 13.5 | | | | | cytoplasm | protein folding | LFVTNDAATILR |
| gil57528682 | thiosulfate sulfurtransferase | 33385.8 | R | 10.6 | | | 13.3 | | | mitochondrion | cyanate catabolism, iron-sulfur cluster assembly | YLGTQPEPDAVGLDSGHIR |
| gil82965326 | PREDICTED: similar to Peroxiredoxin 1 (Thioredoxin peroxidase 2) (Thioredoxin-dependent peroxide re | 22248.4 | O | | 13.2 | | | | | | | LVQAFQFTDK |
| gil82950867 | PREDICTED: similar to Tubulin beta chain (T beta-15) | 38644.1 | M | | 13.1 | | | | | | | SGPFGQIFR |

| | | | | | | | | | | | | |
|---|---|---|---|---|---|---|---|---|---|---|---|---|
| gil56605990 | leucine-rich protein 157 | 156553.5 | R | 13.0 | | | | | | cytoplasm, cytoskeleton, mitochondrion, nucleus | mitochondrion transport along microtubule | SSLSSSSPSAGDTVTEK |
| gil50263048 | glycoprotein M6B isoform 1 | 36195.7 | H | | | | 12.9 | | | membrane | nervous system development | QYGIIPWNAFPGK |
| gil82892852 | PREDICTED: similar to tubulin, beta 5 | 40200.8 | M | | | 0.9 | 12.8 | | 11.5 | | | NSSYFVEWIPNDVK |
| gil113205496 | hypothetical protein LOC311254 | 52927.0 | R | | | | 12.7 | | | | | LTEDEEGNPQVR |
| gil13384916 | ATPase, H+ transporting, V1 subunit C, isoform 1 | 43933.9 | M | 12.7 | | | | | | cytoplasm, | ATP synthesis coupled proton transport, ion transport | GVTQIDNDLK |
| gil28173550 | cell division cycle 10 homolog | 50617.0 | M | | 12.3 | | | 5.9 | | synaptosome, | cell cycle | FEDYLNAESR |
| gil13195624 | NADH dehydrogenase (ubiquinone) 1 alpha subcomplex 10 | 40577.7 | M | 4.4 | | | 5.5 | 12.3 | | mitochondrion, | nucleotide and nucleic acid metabolism | VITVDGNICSGK |
| gil90577179 | septin 5 | 43864.3 | R | 12.3 | | | | 7.3 | | membrane, cytoplasm, synaptosome, | regulation of exocytosis | INQTVEILK |
| gil109486355 | PREDICTED: similar to IQ motif containing with AAA domain | 140794.4 | R | 12.2 | | | | | | | | KELEMEIR |
| gil31980726 | phosphoglucomutase 2 | 61478.7 | M | 12.2 | 7.6 | | | | | | | QEATLVVGGDGR |
| gil400626 | Sodium- and chloride-dependent GABA transporter 3 | 69914.3 | R | 11.9 | | | | | | membrane, | neurotransmitter transport, transport | GDGTISAITEK |
| gil40018616 | chaperonin containing TCP1, subunit 3 (gamma) | 60608.4 | R | 11.8 | | | | | | cytoplasm, | chaperonin-mediated tubulin folding | TLIQNCGASTIR |
| gil82907579 | PREDICTED: similar to Peptidyl-prolyl cis-trans isomerase A (PPIase) (Rotamase) (Cyclophilin A) (Cy | 29249.1 | O | | | | | | 11.7 | | | IIPGFMCQGGDFTR |
| gil68341941 | karyopherin alpha 5 (importin alpha 6) | 60260.9 | R | | | 0.1 | | 11.6 | | | | RNVSLPR |
| gil5902122 | spectrin, beta, non-erythrocytic 2 | 271127.1 | H | 4.7 | 11.6 | | | | | cytoskeleton, membrane, , intracellular, nucleus | barbed-end actin filament capping, vesicle-mediated transport | VHLENMGSHDIVDGNH R |
| gil29336093 | tropomyosin 3, gamma isoform 2 | 29016.7 | R | | | | | | 11.5 | cytoplasm, | regulation of muscle contraction | EQAEAEVASLNR |
| gil124244033 | microtubule-associated protein 1 A | 325756.0 | M | | | | 11.4 | 3.9 | | cytoplasm, cytoskeleton, | sensory perception of sound | AVLDALLEGK |
| gil82884333 | PREDICTED: similar to Spectrin alpha chain, brain (Spectrin, non-erythroid alpha chain) (Alpha-II s | 71479.2 | O | 11.3 | | | | | | | | LIQNNHYAMEDVATR |
| gil31982561 | reticulon 1 isoform RTN1-A | 83520.6 | M | | | | 11.2 | 1.0 | | endoplasmic reticulum, membrane, | biological_process | HQAQVDQYLGLVR |
| gil6681764 | NADH dehydrogenase (ubiquinone) 1 alpha subcomplex, 9, 39kDa | 42482.6 | H | | | | | 11.1 | | mitochondrion, | sodium ion transport | MGSQVIIPYR |
| gil109483639 | PREDICTED: similar to Myosin-5C (Myosin Vc) | 234186.2 | R | 11.1 | | | | | | | | NQSIIVSGESGAGK |
| gil109505063 | PREDICTED: similar to actinin alpha 2 | 139682.8 | R | 1.8 | | | | | 11.0 | | | EGLLLWCQR |
| gil4885393 | epsilon globin | 16192.5 | H | | | | 10.3 | | | cytoplasm | oxygen transport, transport | LLVVYPWTQR |
| gil31543797 | synaptotagmin 2 | 47218.2 | M | 10.3 | | | | | | membrane, synapse | transport | IFVGSNATGTELR |
| gil12018252 | transketolase | 71141.3 | R | 9.9 | 6.6 | | | | | endoplasmic reticulum, peroxisome | regulation of growth | LGQSDPAPLQHQVDVYQ K |
| gil6981462 | acyl-CoA hydrolase | 37537.2 | R | | | | | 9.6 | | cytoplasm | fatty acid catabolism | VLEVPPIVYLR |
| gil82879408 | PREDICTED: similar to Rab-related GTP-binding protein | 40607.4 | M | 9.5 | | | | | | | | MLAEDELR |
| gil7657583 | solute carrier family 25 (mitochondrial carrier, adenine nucleotide translocator), member 13 | 74419.8 | O | | 9.5 | | | | | membrane, mitochondrion | aspartate transport, transport | GLLPQLLGVAPEK |
| gil8393180 | cytochrome c oxidase subunit IV isoform 1 | 19502.1 | R | | | | | | 9.5 | membrane, mitochondrion | | SEDYALPSYVDR |
| gil4502107 | annexin 5 | 35914.4 | H | | | | 9.5 | | | cytoplasm, intracellular | anti-apoptosis, blood coagulation | SEIDLFNIR |
| gil124244102 | ATPase, H+ transporting, lysosomal V0 subunit a isoform 2 | 142771.9 | R | | | | 9.3 | | | cytoplasm, extracellular region, membrane | immune response | LTFLNSFK |
| gil13592152 | glutathione S-transferase, mu type 3 | 25664.0 | R | | 0.7 | | | | 9.3 | | | LCYNPDFEK |
| gil27552760 | translocase of outer mitochondrial membrane 70 homolog A | 67546.9 | M | 8.9 | | | | | | membrane, mitochondrion | | AAAFEQLQK |
| gil109503054 | PREDICTED: similar to oxoglutarate dehydrogenase-like | 123543.0 | R | 8.8 | | | | | | | | SSPYPTDVAR |

| | | | | | | | | | | | | |
|---|---|---|---|---|---|---|---|---|---|---|---|---|
| gil57114330 | ubiquinol-cytochrome c reductase, Rieske iron-sulfur polypeptide 1 | 29427.2 | R | | | | | | 8.6 | membrane, mitochondrion, protein complex | electron transport | VPDFSDYR |
| gil6981668 | tenascin R | 149276.5 | R | | | | 8.6 | | | cell surface, extracellular matrix, membrane | cell adhesion, extracellular matrix organization and biogenesis | SSLTSTIFTTGGR |
| gil13540707 | profilin 2 | 16103.9 | R | | | | | | 8.5 | cytoskeleton | regulation of actin filament polymerization | SQGGEPTYNVAVGR |
| gil21361370 | brain glycogen phosphorylase | 96634.5 | H | | | | 8.5 | | | | glycogen catabolism | GLAGLGDVAEVR |
| gil6981716 | mitogen activated protein kinase kinase kinase 12 | 96247.3 | R | | | 6.2 | 8.5 | | | cytoplasm, cytoplasm, membrane | histone phosphorylation, JNK cascade | LEEELVMRR |
| gil30520131 | solute carrier family 6 (neurotransmitter transporter, GABA), member 1 | 66957.1 | M | 8.3 | | | | | | axon, membrane | neurotransmitter transport | VADGQISTEVSEAPVASD KPK |
| gil62662345 | PREDICTED: hypothetical protein XP_577496 | 71469.2 | R | 8.3 | | | | | | | | QTVAVGVIK |
| gil8393358 | fumarate hydratase 1 | 54429.0 | R | 8.2 | | | | | | cytoplasm, mitochondrion | fumarate metabolism | AAAEVNQEYGLDPK |
| gil109465447 | PREDICTED: similar to cytochrome c oxidase, subunit VIb polypeptide 1 | 17710.7 | O | | | | | | 8.2 | | | IAEGTFPGK |
| gil6671672 | capping protein (actin filament) muscle Z-line, alpha 2 | 32946.6 | M | | | | 8.0 | | | cytoskeleton | actin cytoskeleton organization and biogenesis | FTVTPSTTQVVGILK |
| gil109948279 | hypothetical protein LOC653499 | 14944.0 | H | | | | 7.9 | | | | | LDTSEVVFNSK |
| gil94385873 | PREDICTED: similar to Glyceraldehyde-3-phosphate dehydrogenase (GAPDH) | 57373.8 | M | | 7.9 | | 4.1 | | 3.2 | | | VIISAPSADAPMFVMGV NHEK |
| gil54019419 | proteasome (prosome, macropain) subunit, beta type 6 | 25273.4 | R | | | | | 7.8 | | cytoplasm, nucleus, protein complex | ubiquitin-dependent protein catabolism | LAAIQQSGVER |
| gil30023842 | valosin containing protein | 89307.8 | M | 7.8 | | | | 1.9 | 1.0 | cytoplasm, endoplasmic reticulum, microsome, nucleus | caspase activation, ER-associated protein catabolism | LAGESESNLR |
| gil109501680 | PREDICTED: similar to Dihydropyrimidinase-related protein 2 (DRP-2)(Turned on after division, 64 kDa protein) (TOAD-64) (Collapsin response mediator protein 2) (CRMP-2) | 20767.5 | R | 7.8 | | | | | 5.8 | | | EALQNIYDQSDR |
| gil4557515 | damage-specific DNA binding protein 2 (48kD) | 47833.4 | H | | | | 7.4 | | | nucleus | nucleotide-excision repair | TSEIVLRPRNK |
| gil109465364 | PREDICTED: similar to Glutathione S-transferase Mu 6 (GST class-mu 6) (Glutathione-S-transferase class M5) | 25495.0 | R | 7.3 | 1.8 | | | | | | | ITQSNAILR |
| gil16924002 | DJ-1 protein | 19961.4 | R | | | | | 1.5 | 7.3 | nucleus | adult locomotory behavior, synaptic transmission, dopaminergic | DVVICPDTSLEEAK |
| gil4885377 | H1 histone family, member 3 | 22336.3 | H | 7.3 | 6.8 | | | | | chromosome, nucleus | nucleosome assembly | ALAAAGYDVEK |
| gil4826655 | calbindin 1 | 30006.0 | H | | | | | | 7.0 | | | TFVDQYGQR |
| gil6980956 | glutamate dehydrogenase 1 | 61377.3 | R | 7.0 | 2.4 | | | | | mitochondrion | long-term memory, transmembrane receptor protein tyrosine kinase signaling pathway | NYTDNELEK |
| gil32455244 | beta isoform of regulatory subunit A, protein phosphatase 2 isoform b | 73538.2 | H | | 7.0 | | | | | cytoplasm, membrane, cytoskeleton, mitochondrion, nucleus | ceramide metabolism, inactivation of MAPK activity, induction of apoptosis, negative regulation of cell growth | TSACGLFSVCYPR |
| gil42821116 | neural cell adhesion molecule 2 | 93123.4 | R | 6.9 | | | | | | extracellular region, membrane | axonal fasciculation, cell adhesion, sensory perception of smell | IIELSQTTAK |
| gil13929002 | phosphofructokinase, muscle | 85505.7 | R | 6.8 | | | 2.3 | | | cytoplasm, | regulation of glycolysis | LPLMECVQVTK |
| gil11560002 | amphiphysin 1 | 74832.0 | R | 6.7 | | | | | | cytoskeleton, cytoplasm | learning, regulation of GTPase activity, synaptic vesicle endocytosis | EATEDVAPQGPAGEK |
| gil33414505 | chaperonin subunit 4 (delta) | 58062.8 | R | 6.7 | | | | | | cytoplasm, | chaperone cofactor-dependent protein folding, chaperonin-mediated tubulin folding | LVIEEAER |
| gil16924010 | septin 2 | 41566.3 | R | | | | | 6.6 | | cytoplasm, synaptosome | neurite development | LTVVDTPGYGDAINSR |
| gil21264345 | peripherin | 53618.4 | H | 6.6 | | | | 0.7 | | cytoskeleton, | | AQYESIAAK |

| | | | | | | | | | | | | |
|---|---|---|---|---|---|---|---|---|---|---|---|---|
| gi\|62660026 | PREDICTED: similar to glyceraldehyde-3-phosphate dehydrogenase | 40957.0 | R | | | | 6.6 | | | | | LVINGKPITIFQER |
| gi\|35215309 | neurofascin | 137889.2 | M | 6.5 | | | | | | axon, membrane | axon guidance | TSGAPPESNPSDVK |
| gi\|78365255 | dihydrolipoamide S-acetyltransferase (E2 component of pyruvate dehydrogenase complex) | 67123.4 | O | | | 1.3 | 6.4 | | | mitochondrion, cytoplasm | acetyl-CoA biosynthesis | AAPAAAAAAPPGPR |
| gi\|32483416 | neurofilament, heavy polypeptide 200kDa | 111770.8 | H | 6.2 | | | | | | axon, cytoskeleton, chromosome, nucleus | nervous system development, nucleosome assembly | SAMGELYER |
| gi\|9507177 | transcytosis associated protein | 107158.8 | R | 6.2 | | | | | | cytoplasm, Golgi apparatus, microsome | ER to Golgi vesicle-mediated transport | GVMGGQSAGPQHTEAE TIQK |
| gi\|4505357 | NADH dehydrogenase (ubiquinone) 1 alpha subcomplex, 4, 9kDa | 9363.9 | H | | | | | | 6.1 | mitochondrion, | | FYSVNVDYSK |
| gi\|82907477 | PREDICTED: similar to translocase of outer mitochondrial membrane 7 homolog | 6200.4 | M | 6.0 | | | | | | | | LQQLFR |
| gi\|109486463 | PREDICTED: similar to Ras-related protein Rab-12 | 45201.4 | R | | | | 5.1 | 5.9 | 4.5 | | | LQIWDTAGQER |
| gi\|57222274 | PHD finger protein 8 | 102075.5 | M | | | 5.8 | | | | | | HLCTTDLFKFPSFETICW HVGK |
| gi\|31981350 | hypothetical protein LOC76820 | 37318.7 | M | 5.7 | | | | | | | | VMLETPEYR |
| gi\|21704242 | vesicle-associated calmodulin-binding protein | 54785.5 | M | 5.7 | 5.1 | | | | | | | PFGCVTLGDK |
| gi\|4757900 | calreticulin precursor | 48111.8 | H | 5.4 | | | | | | cytoplasm, endoplasmic reticulum | calcium ion homeostasis, protein export from nucleus, protein folding | HEQNIDCGGGYVK |
| gi\|13786204 | voltage-dependent anion channel 3 | 30778.4 | R | | 5.4 | | | | | mitochondrion | adenine transport, behavioral fear response, learning, nerve-nerve synaptic transmission | LTVDTIFVPNTGK |
| gi\|30065645 | protein phosphatase 2A, regulatory subunit B' isoform a | 40641.4 | H | | | | | | 5.2 | soluble fraction | protein amino acid dephosphorylation | FGSLLPIHPVTSG |
| gi\|14389299 | vimentin | 53700.0 | R | | 5.0 | | | 4.6 | | cytoplasm, cytoskeleton, nucleus | cell motility, intermediate filament-based process | EEAESTLQSFR |
| gi\|109460227 | PREDICTED: similar to Alpha-centractin (Centractin)(Centrosome-associated actin homolog) (Actin-RPV) (ARP1) | 51309.1 | O | 4.9 | | | | | | | | AGFAGDQIPK |
| gi\|4505937 | polymerase (DNA directed), gamma | 139473.4 | H | | | | | 4.8 | | mitochondrion | base-excision repair, gap-filling, DNA-dependent DNA replication | HCLEQGK |
| gi\|4758986 | RAB11B, member RAS oncogene family | 24558.6 | H | | 4.8 | | | | | membrane | protein transport, small GTPase mediated signal transduction | STIGVEFATR |
| gi\|62645331 | PREDICTED: similar to Nck-associated protein 1 (NAP 1) (p125Nap1) (Membrane-associated protein HEM-2) | 141549.4 | R | 4.8 | | | | | | | | LSSVDSVLK |
| gi\|47132509 | septin 5 | 42848.7 | M | | | | | 4.7 | | membrane, cytoplasm, synaptosome | cell cycle, regulation of exocytosis | MESPIPILPLPTPDAETEK |
| gi\|24308229 | protein kinase C and casein kinase substrate in neurons 1 | 50933.8 | H | | 4.6 | | | | | cytoplasm | endocytosis | GPQYGSLER |
| gi\|16758270 | F-box only protein 2 | 33679.0 | R | | | | 4.5 | | | cytoplasm | ER-associated protein catabolism, glycoprotein catabolism | ELVDGAPLWLLK |
| gi\|31317307 | proprotein convertase subtilisin/kexin type 9 preproprotein | 74239.1 | H | | | | | 4.4 | | | cholesterol metabolism | MSGDLLELALK |
| gi\|9507125 | synuclein, alpha | 14506.2 | R | | | | | | 4.4 | cytoplasm, synaptosome | anti-apoptosis, central nervous system development, dopamine biosynthesis, dopamine metabolism | EGVVHGVTTVAEK |
| gi\|31981282 | glyoxalase 1 | 20826.4 | M | | 4.3 | | | | | cytoplasm | anti-apoptosis | DFLLQQTMLR |
| gi\|109467991 | PREDICTED: similar to Serine/threonine-protein phosphatase 2A regulatory subunit B (PP2A, subunit B, PR53 isoform) (Phosphotyrosyl phosphatase activator) (PTPA) | 36486.0 | R | | 4.3 | | | | | | | LVALLDTLDR |
| gi\|116805788 | cytoplasmic FMR1 interacting protein 2 | 145599.0 | H | 4.3 | | | | | | cytoplasm, synaptosome | | AVGPSSTQLYMVR |

| | | | | | | | | | | | | |
|---|---|---|---|---|---|---|---|---|---|---|---|---|
| gil5729850 | guanine nucleotide binding protein (G protein), alpha inhibiting activity polypeptide 3 | 40506.3 | O | 4.2 | | | | | | | G-protein coupled receptor protein signaling pathway, negative regulation of adenylate cyclase activity | EIYTHFTCATDTK |
| gil68163411 | activated leukocyte cell adhesion molecule | 65061.1 | H | 4.2 | | | | | | membrane | | SSNTYTLTDVR |
| gil32401457 | opsin 5 | 59284.5 | R | | 0.7 | | | | 4.1 | | | MALNHTALPQDER |
| gil30409998 | solute carrier family 25 (mitochondrial carrier; adenine nucleotide translocator), member 31 | 35265.4 | O | | | | 4.0 | | | flagellum, membrane, mitochondrion | | GNLANVIR |
| gil110347439 | zinc finger protein 225 | 82382.9 | H | | 3.9 | | | | | intracellular, nucleus | regulation of transcription, DNA-dependent, transcription | VHMGEK |
| gil62661476 | PREDICTED: similar to N-copine | 219591.5 | R | | 3.9 | | | | | | | FLVYDYDSSGK |
| gil70906474 | Ca<2+>dependent activator protein for secretion | 153906.3 | M | 3.8 | | | | | | cytoplasm, membrane, synapse | catecholamine secretion, exocytosis, protein transport, transport | AGGGRPSSPSPSVVSEK |
| gil114326502 | paxillin isoform beta | 64434.8 | M | | | | 3.8 | | | cytoskeleton, membrane, lamellipodium | | AGPLVK |
| gil110625946 | kinesin family member 2B | 79529.1 | M | | | | 3.7 | | | cytoskeleton | microtubule-based movement | KSQVE |
| gil62666823 | PREDICTED: similar to prohibitin | 30973.2 | R | 3.7 | | | | | | | | EFTEAVEAK |
| gil9506941 | oligodendrocyte myelin glycoprotein | 49252.0 | M | 3.5 | | | | | | extracellular matrix, membrane | cell adhesion | LESLPAQLPR |
| gil82697315 | rap2 interacting protein x isoform 1 | 70564.8 | H | 3.5 | | | | | | | | TAEGQALSEAR |
| gil4503725 | FK506-binding protein 1A | 11943.1 | H | | | | | | 3.4 | | positive regulation of I-kappaB kinase/NF-kappaB cascade, protein folding | GWEEGVAQMSVGQR |
| gil94388698 | PREDICTED: TBP-interacting protein isoform 1 | 164765.0 | M | 1.9 | | | 3.4 | | | | | HTVDDGLDIR |
| gil109489185 | PREDICTED: similar to angiotensin I converting enzyme(peptidyl-dipeptidase A) 1 isoform 1 | 90074.4 | R | | | | 3.4 | | | | | SQFMLYFGTR |
| gil109474831 | PREDICTED: similar to Protein KIAA1045 | 51326.4 | R | 1.3 | | | | | 3.3 | | | EAEASAPGVQEESSSNR |
| gil62945366 | hypothetical protein LOC498407 | 33397.6 | R | | 3.3 | | | | | | | GGGGPGGEQETQELASK |
| gil62652199 | PREDICTED: similar to Inner nuclear membrane protein Man1 (LEM domain containing protein 3) | 76201.9 | O | | 3.2 | | | | | | | TAVAPLPR |
| gil29789257 | RAB5C, member RAS oncogene family | 25335.7 | M | 3.2 | 1.6 | | | | | cytoplasm, membrane | regulation of endocytosis, small GTPase mediated signal transduction, transport | GVDLQESNPASR |
| gil109475709 | PREDICTED: similar to loss of heterozygosity, 11, chromosomal region 2, gene A homolog | 134608.0 | R | | | | 3.2 | | | | | QPLRQGHPR |
| gil6981420 | pancreatic trypsin 1 | 25942.7 | R | 3.0 | | | 3.2 | | | extracellular region, mitochondrion | calcium ion binding, peptidase activity, trypsin activity | LGEHNINVLEGDEQFINAAK |
| gil12621120 | sideroflexin 3 | 35410.6 | R | | | | | | 3.1 | membrane, mitochondrion | iron ion transport | ELQVGIPVTDEAGQR |
| gil62990176 | thyroid stimulating hormone receptor | 86527.4 | M | | | | 3.1 | | | membrane | adult locomotory behavior, G-protein coupled receptor protein signaling pathway, positive regulation of body size, regulation of locomotion | NPQYNPRDK |
| gil5729804 | nudix-type motif 3 | 19458.7 | H | | | | | | 2.9 | intracellular | cyclic-nucleotide-mediated signaling, diadenosine polyphosphate catabolism, regulation of RNA export from nucleus | LVGIFENQER |
| gil112382252 | spectrin, beta, non-erythrocytic 1 isoform 2 | 251263.2 | H | 2.7 | | | | | | cytoskeleton, membrane | barbed-end actin filament capping | ALVADSHPESER |
| gil52317184 | olfactory receptor, family 10, subfamily X, member 1 | 36421.1 | H | | | | | 2.7 | | membrane | G-protein coupled receptor protein signaling pathway, sensory perception of smell | IPSAEGKQK |
| gil30519995 | sideroflexin 5 | 37304.9 | M | | | | | | 2.6 | membrane, mitochondrion | cation transport, ion transport | EAVQLLEDYK |
| gil7661966 | mediator of DNA damage checkpoint 1 | 226554.8 | H | 2.5 | | | | | | intracellular, nucleus | cell cycle, DNA repair | VEPAGRSR |
| gil34868383 | PREDICTED: similar to Mrpl50 protein | 18307.5 | R | | | 2.5 | | | | | | AALCVCR |
| gil63475897 | PREDICTED: similar to ADP/ATP translocase 2 (Adenine nucleotide translocator 2) (ANT 2) (ADP,ATP ca | 32854.9 | O | | | | | 2.4 | | | | DFLAGGVAAAISK |

| | | | | | | | | | | | | |
|---|---|---|---|---|---|---|---|---|---|---|---|---|
| gil82881104 | PREDICTED: similar to transmembrane protease, serine 9 | 68005.0 | M | | 2.4 | | | | | | | SHQEAPR |
| gil94388156 | PREDICTED: similar to Pre-mRNA splicing factor PRP17 (Cell division cycle 40 homolog) isoform 7 | 65789.4 | O | | | | 2.3 | | | | | QEEEKPGEEK |
| gil82881174 | PREDICTED: similar to Peroxiredoxin 2 (Thioredoxin peroxidase 1) (Thioredoxin-dependent peroxide re | 21891.1 | O | | | | | 2.2 | | | | NDEGIAYR |
| gil42544243 | dynamin 3 | 97034.6 | H | | 2.2 | | | | | intracellular, ribosome | endocytosis, protein biosynthesis | HIFALFNTEQR |
| gil6912464 | latrophilin 2 precursor | 157077.1 | H | | | | 2.0 | | | membrane | G-protein coupled receptor protein signaling pathway, neuropeptide signaling pathway | LGADFIGR |
| gil47577367 | olfactory receptor Olr436 | 34781.9 | R | | | | | 2.0 | | membrane | detection of chemical stimulus during sensory perception of smell, G-protein coupled receptor protein signaling pathway | MSSAAGRK |
| gil4502505 | complement component 4 binding protein, beta chain isoform 1 precursor | 28338.4 | H | | | | | 1.9 | | extracellular region | blood coagulation, complement activation | ALLAFQESK |
| gil61966807 | hypothetical protein LOC401233 | 37258.6 | H | | | 1.9 | | | 1.4 | | | KSLALSPR |
| gil47778923 | cytochrome b5 reductase b5R.2 isoform 1 | 31438.4 | H | | | | 1.9 | | | membrane, soluble fraction | | LGYTQDMIFTY |
| gil66792792 | branched chain aminotransferase 1, cytosolic isoform 1 | 49868.4 | M | 1.8 | | | | | | cytoplasm | branched chain family amino acid biosynthesis, branched chain family amino acid catabolism | LTDIQYGR |
| gil6996005 | dynamin 1-like protein isoform 1 | 81840.1 | H | 1.7 | | | | | | endoplasmic reticulum, Golgi apparatus, membrane | endocytosis, mitochondrial membrane organization and biogenesis | AEELLAEEK |
| gil62643182 | PREDICTED: similar to AFL095Wp | 187628.8 | R | 1.7 | | | | | | | | EGLELPEDEEEK |
| gil82942324 | PREDICTED: hypothetical protein XP_924279 | 7348.4 | M | | | | | 1.6 | | | | AVNAFCPGSR |
| gil61098157 | hypothetical protein LOC242800 | 60220.4 | M | | | | 1.6 | | | | | VATSGCRLH |
| gil83012875 | PREDICTED: similar to Peroxiredoxin 6 (Antioxidant protein 2) (1-Cys peroxiredoxin) (1-Cys PRX) (Ac | 24885.0 | O | | 1.6 | | | | | | | VVFIFGPDK |
| gil89043015 | PREDICTED: similar to coiled-coil domain containing 40 isoform 12 | 107087.0 | H | | 1.6 | | | | | | | DEAHRAVLEALR |
| gil22164792 | mitochondrial ribosomal protein L12 | 21694.8 | M | | | 1.6 | | | | intracellular, mitochondrion, ribosome | protein biosynthesis | MLPVAASRCLWGPR |
| gil4502365 | bagpipe homeobox 1 | 34792.4 | H | | | | 1.6 | | | nucleus | skeletal development, transcription from RNA polymerase II promoter | TEDDGVGPR |
| gil21699074 | zinc finger protein 688 isoform a | 30555.4 | H | | | 1.6 | | | | intracellular, nucleus | regulation of transcription, DNA-dependent, transcription | HVCTDCGR |
| gil109472087 | PREDICTED: similar to Glycyl-tRNA synthetase | 87626.7 | R | | 1.6 | | | | | | | AEVSELPSVVR |
| gil4758988 | RAB1A, member RAS oncogene family | 22663.4 | H | | | | 1.5 | | | cytoplasm, endoplasmic reticulum, intracellular, membrane | ER to Golgi vesicle-mediated transport, protein transport, small GTPase mediated signal transduction | EFADSLGIPFLETSAK |
| gil4504483 | hypoxanthine phosphoribosyltransferase 1 | 24563.6 | H | 1.5 | | | | | | cytoplasm | behavior, hypoxanthine metabolism, nucleoside metabolism, positive regulation of dopamine metabolism | SYCNDQSTGDIK |
| gil82881242 | PREDICTED: hypothetical protein XP_909678 | 9476.4 | M | | | 1.5 | | | | | | TQNSSFSENPR |
| gil62650368 | PREDICTED: similar to tyrosine kinase | 387965.1 | R | | 1.5 | | | | | | | DFDLNRVPSAR |
| gil65301141 | PH domain and leucine rich repeat protein phosphatase-like | 146658.0 | H | | | | | | 1.5 | cytoplasm | | GSCFGIRR |
| gil62667525 | PREDICTED: centrin 2 | 38009.0 | R | | | | 1.5 | | | | | KTSLY |
| gil24308191 | p21-activated kinase 7 | 80694.0 | H | | | | 1.5 | | | | protein amino acid phosphorylation | TAGTSGCSK |
| gil86792778 | dipeptidyl-peptidase 6 isoform 2 | 91283.9 | H | | | | | 1.4 | | membrane | proteolysis | VVSTGK |
| gil4502995 | phakinin | 45851.2 | H | | | | 1.3 | | | cytoskeleton, membrane | response to stimulus, visual perception | AHLLAR |

| | | | | | | | | | | | | |
|---|---|---|---|---|---|---|---|---|---|---|---|---|
| gil82929824 | PREDICTED: similar to U2 small nuclear ribonucleoprotein B | 22118.9 | M | | | | 1.3 | | | | | AVNYQSTCPNNCFK |
| gil109499524 | PREDICTED: similar to SEPT11 protein | 65069.2 | R | | | | 1.2 | | | | | FESDPATHNEPGVR |
| gil94393568 | PREDICTED: similar to Brain-enriched guanylate kinase-associated protein isoform 3 | 67188.7 | M | 1.2 | | | | | | | | SGPRPPYK |
| gil47717102 | ATPase, H+ transporting, lysosomal 50/57kDa, V1 subunit H isoform 2 | 55847.1 | H | 1.2 | | | | | | cytoplasm, membrane | ATP hydrolysis coupled proton transport | QLQSEQPQTAAAR |
| gil82890177 | PREDICTED: similar to WD repeat domain 49 | 47029.6 | M | | 1.2 | | | | | | | IHISPI |
| gil82932924 | PREDICTED: similar to Absent in melanoma 1 protein | 248690.2 | M | | | 1.2 | | | | | | APAAADSGEEAGR |
| gil38679960 | acetyl-Coenzyme A carboxylase alpha isoform 1 | 269827.3 | H | | | | | | 1.1 | | fatty acid biosynthesis | GHMKAVVMDLLR |
| gil133778965 | coiled-coil domain containing 67 | 67436.4 | M | | | | | 1.1 | | | | LTSNFEKLR |
| gil33942124 | defensin beta 34 | 9261.4 | M | | | | | 1.0 | | extracellular region | defense response | NLKCCVTVQSCGR |
| gil83019634 | PREDICTED: similar to MOs against decapentaplegic homolog 5 (SMAD 5) (MOs against DPP homol | 46649.0 | O | | 1.0 | | | | | | | SRFCLGLLINVNR |
| gil6678768 | myristoylated alanine rich protein kinase C substrate | 29643.8 | M | 1.0 | | | | | | cytoplasm, nucleus, membrane | | AEDGAAPSPSSETPK |
| gil8393274 | dentatorubral pallidoluysian atrophy | 124702.3 | R | | | | | | 1.0 | cytoplasm, nucleus | negative regulation of transcription from RNA polymerase II promoter, regulation of neuron differentiation, toxin metabolism | GGAAASSVGPPSGGK |
| gil118572606 | hemicentin 1 | 613129.4 | H | | 0.9 | | | | | membrane | blood coagulation, visual perception | QNVCRPDQHCKNTR |
| gil109500138 | PREDICTED: similar to echinoderm microtubule associated protein like 5 | 55641.0 | R | | | | | 0.9 | | | | CAAYSPDGEMVAIGMK |
| gil113415244 | PREDICTED: similar to alpha 3 type VI collagen isoform 1 precursor | 177605.8 | H | | 0.8 | | | | | | | VYAIGIK |
| gil88955017 | PREDICTED: hypothetical protein XP_949783 | 34413.8 | H | | | | 0.8 | | | | | QSRCSGEPALHR |
| gil7662006 | PHD finger protein 16 | 93677.0 | H | | | | 0.8 | | | | regulation of transcription, DNA-dependent | ITLK |
| gil38259186 | adiponectin receptor 1 | 42338.4 | M | | | 0.8 | | | | membrane | fatty acid oxidation, hormone-mediated signaling | GSAGAQGNGAPSGNR |
| gil66932916 | mitogen-activated protein kinase 1 | 41363.2 | H | | | | | | 0.8 | | cell cycle, chemotaxis, induction of apoptosis, synaptic transmission | FDMELDDLPK |
| gil6806905 | CD7 antigen | 23137.9 | M | | | | | | 0.8 | membrane | immune response | LTIASEGDSVNITCSTRG HLEGILMK |
| gil24638433 | nicastrin precursor | 78361.5 | H | | | 0.7 | | | | endoplasmic reticulum, Golgi apparatus, membrane | amyloid precursor protein catabolism, membrane protein ectodomain proteolysis, Notch receptor processing | LLSFCVLLAGLCR |
| gil5453764 | nel-like 1 precursor | 89545.9 | H | | 0.7 | | | | | | cell adhesion, nervous system development | MADGQWHK |
| gil82901693 | PREDICTED: similar to tubulin, alpha 1 | 26995.9 | M | | 0.7 | | | | | | | YMACCLLYRCNGVPK |
| gil94398391 | PREDICTED: similar to ribonuclease III | 159181.4 | M | | | | 0.7 | | | | | GINTLINIMSR |
| gil62652681 | PREDICTED: similar to Zinc finger protein 7 (Zinc finger protein KOX4) (Zinc finger protein HF.16) | 80600.1 | O | | | 0.6 | | | | | | KPYGCEECGK |
| gil7657395 | photoreceptor-specific nuclear receptor isoform b | 44663.4 | H | | | | | 0.6 | | nucleus | phototransduction, transcription from RNA polymerase II promoter, visual perception | AHRNQCACR |
| gil109458660 | PREDICTED: similar to ATP binding domain 3 | 44135.5 | R | | | | | | 0.6 | | | MPAPTCFSCHK |
| gil27545326 | SWI/SNF related, matrix associated, actin dependent regulator of chromatin, subfamily b, member 1 i | 44113.2 | O | | | | | | 0.6 | chromosome, nucleus | cell cycle, chromatin remodeling, DNA integration, regulation of transcription from RNA polymerase II promoter | MMMMALSKTFGQKPVK |
| gil62641476 | PREDICTED: CD 81 antigen | 25887.1 | R | | | | 0.6 | | | | | IDELFSGK |
| gil41350216 | beta-1,4-mannosyltransferase | 52483.9 | H | | | | 0.6 | | | endoplasmic reticulum, membrane | biosynthesis | LGSMHSPFR |
| gil62644075 | PREDICTED: similar to mitochondrial tryptophanyl tRNA synthetase 2 | 72431.8 | R | | | | 0.6 | | | | | MSKSDPDK |
| gil6981000 | guanylate cyclase 2C | 123387.7 | R | | | | | 0.6 | | brush border, membrane | cGMP biosynthesis | NLEHLVEER |
| gil109469003 | PREDICTED: hypothetical protein | 109614.0 | R | | | | 0.5 | | | | | SPSTCSPCQRGR |

| | | | | | | | | | | | | |
|---|---|---|---|---|---|---|---|---|---|---|---|---|
| gil62658791 | PREDICTED: tyrosylprotein sulfotransferase 1 (predicted) | 89470.0 | R | 0.5 | | | | | | | | SGDLPPREHVR |
| gil12232413 | zinc finger CCCH-type domain containing 1 | 79013.1 | H | | 0.4 | | | | | nucleus | protein amino acid ADP-ribosylation | FMVYGACK |
| gil42656882 | PREDICTED: hypothetical protein XP_379234 | 10508.1 | H | | | | 0.4 | | | | | SIWSHLAALGCDR |
| gil116805342 | human immunodeficiency virus type I enhancer binding protein 1 | 296735.0 | H | | | | | | 0.4 | intracellular, nucleus | regulation of transcription, DNA-dependent, transcription | AHSK |
| gil6981168 | lipoprotein lipase | 53048.9 | R | | | | | | 0.4 | extracellular matrix, extracellular region, membrane | circulation, fatty acid metabolism | CNNVGYEINKVR |
| gil23618879 | natural killer cell protease 7 | 26871.1 | R | | | | 0.4 | | | cytoplasm | apoptosis, proteolysis | VILGAHNLHVQEK |
| gil51538581 | chromodomain protein, Y-linked, 1B isoform b | 61977.8 | H | | | 0.4 | | | | | | IWSSAQGIESMLK |
| gil82943718 | PREDICTED: similar to epsin isoform 4 | 46433.9 | M | | | | | | 0.3 | | | TQINNYVMEKSQK |
| gil66730329 | hypothetical protein LOC499418 | 38116.8 | R | | | | 0.3 | | | | | RLLSESCQDSCGR |
| gil113419175 | PREDICTED: similar to Serine/threonine-protein kinase MARK2 (MAP/microtubule affinity-regulating kinase 2) (ELKL motif kinase) (EMK1) (PAR1 homolog) | 27836.6 | O | | 0.2 | | | | | | | FPQGICSKGNSLRPR |
| gil62657218 | PREDICTED: similar to DEAD box polypeptide 42 protein | 102004.5 | R | | | | | 0.2 | | | | GGGRHSHGDSGNR |
| gil6755232 | protein tyrosine phosphatase, non-receptor type 13 | 270837.0 | M | | | | | 0.2 | | cytoplasm, nucleus | | CDPK |
| gil109503581 | PREDICTED: similar to ATPase, class VI, type 11A | 135636.9 | R | | | | | | 0.2 | | | VVCSR |
| gil22094119 | myosin XVIIIa | 230764.8 | M | | | | | | 0.2 | cytoskeleton, myosin | biological_process | GSRLLDAMR |
| gil62078893 | hypothetical protein LOC314432 | 117713.0 | R | | 0.2 | | | | | | | SPPAVQQDNVDEDLIR |
| gil110431378 | utrophin | 392261.4 | M | | | | | | 0.2 | membrane, synapse | adult somatic muscle development | QVGKALEK |
| gil82901669 | PREDICTED: similar to WW domain binding protein 11 (predicted) | 29503.1 | M | | 0.1 | | | | | | | LQMAGQEIPDEGR |
| gil21955148 | cytochrome P450, 3a18 | 57269.6 | R | | | | 0.1 | | | endoplasmic reticulum, membrane, microsome | | AITMSEDEEWKR |
| gil76096352 | hypothetical protein LOC361571 | 85360.7 | R | | | | | | 0.0 | | | GLDGAVDMGARGAAAR |

H; Homo sapiens, R; Rattus norvegicus, M; Mus musculus, O; Others

Thal.; Thalamus, Hipp.; Hippocampus, Fron.;Frotal cortex, Pari.;Parietal cortex, Occi.; Occipital cortex, Amyg.;Amygdala

Tax.; Taxonomy
