# Supplementary material for: Proteomic analysis of proteins expressing in regions of rat brain by a combination of SDS-PAGE with nano-liquid chromatography-quadrupole-time of flight tandem mass spectrometry
Source: Proteome Sci. 2010 Jul 27;8:41. doi: 10.1186/1477-5956-8-41 (PMC2918549; doi:10.1186/1477-5956-8-41)
Supplement: Additional file 2 — Table S2. Lists of ingenuity networks generated by proteins identified in each region of rat brain. [file 1477-5956-8-41-S2.PDF]

Table S2. Lists of ingenuity networks generated by proteins identified in each region of rat brain

Thalamus

ID	Molecules in Network	Score	Focus Molecules	Top Functions
1	*Actin, ACTR1A, ADCY, AMPH, CRHR1, CYFIP2 (includes EG:26999), DCTN1, DUOX2, E2f, EZR, F Actin, FSH, G protein alpha, GAD, GAD1, GAD2, GNAI3, GNAZ, GRIK1, GSN, KIF5B, Lh, MAG, MARCKS (includes EG:4082), MBP, MYO5B, MYO5C, NEFH, NFkB (complex), Pkc(s), PP2A, Rac, Rho gdi, SLC6A1, SPTBN1*	49	22	Amino Acid Metabolism, Molecular Transport, Small Molecule Biochemistry
2	*ACTR3, ARPC5, ATP6V1H, BAT2, BCAT1, C15ORF29, CD46, CHCHD2, CHCHD3, CHD4, DBF4, DHX8, EPCAM, ETFA, HIST2H3C, Histone h3, HNF4A, IMMT, KIF22, LRPPRC, LRRC40, MIR290 (includes EG:100049710), MRPL44, MYC, NDUFS2, NDUFS3, PHB (includes EG:5245), PNKP, PSAT1, SAMM50, SNX25, TOMM70A, TXNDC9, USO1, VEGFA*	35	17	Cardiovascular System Development and Function, Organismal Development, Tumor Morphology
3	*ACADM, ANKS1B, BDNF, beta-estradiol, CALB2, Calbindin, CNTNAP1, DLG2, DOCK7 (includes EG:85440), ERBB2, ETFB, FCGRT, FYN, GAD2, glycosylphosphatidylinositol, GPD1, HDAC6, HECTD1, HPRT1, LHX2, LRP8, MAP2K3, MAP2K6, MAP4K5, MIR103-1 (includes EG:406895), MLXIP, NFASC, OGDHL, SIK1, SLC27A1, SOX4, SOX21, SYNGR3, TNF, YWHAG*	27	14	Cell Death, Cell-To-Cell Signaling and Interaction, Dermatological Diseases and Conditions
4	*ACTN1, Akt, ALCAM, B2m-Mhc1a, CABC1, CABLES2, CADPS, CALR, CANX, CANX-CALR-CD1, Cd1, CD1D-CANX-CALR, CD1D-CANX-CALR-ERp57, DCT, ERK1/2, FCGRT, H2-LD, HLA-B27, ICAM4 (includes EG:3386), IgG, ITGAV, ITGB3, MAG, MAP2K6, MSR1, NCAM2, phosphatidylinositol 4,5-diphosphate, POLD3, PRNP, RAPH1, TACSTD2, TAPBP, TP53, TSHR, TYR*	21	12	Cellular Function and Maintenance, Cell Death, Cell-To-Cell Signaling and Interaction
5	*Ca2+, CACNA1A, CCT2, CCT3, CCT4, CCT5, CCT7, CCT8, CCT6A, CDK19, CTTNBP2, DICER1, DOCK5, EIF2B2, EIF2C4, FZD2, GCN1L1, MAP1A, MED29, PAFAH1B2, PIWIL2, PPP2R4, PPP2R2C, PPP2R2D, PPP4C, SLC2A4, SSSCA1, STK24, STRN, STRN3, SYT2, SYT7, TCP1, TSG101, ZFYVE27*	9	6	Cell Death, Post-Translational Modification, Protein Folding

Hippocampus

ID	Molecules in Network	Score	Focus Molecules	Top Functions
1	*26s Proteasome, Actin, ALDH2, BRF2, Caveolin, CCT8, CTTN, DNM3, DOCK5, Dynamin, ERCC6, ERK1/2, FPR1, GMNN, GNAI2, HSPD1, KEAP1, MCM7, MFN2, MUC2, NFkB (complex), P38 MAPK, PACRG, PACSIN1, PASK, PPP2R4, PPP2R1B, RAGE, RAPGEF3, RELT, RGS3, RGS16, S1PR2, S1PR3, SEPT4*	40	19	Nervous System Development and Function, Cell Morphology, Cellular Development
2	*CCNT2, CD59, CDKN2AIP, CHL1, DLD, DLG4, EPB41L2, FBXO11, FDXR, GRB2, GSN, HSPD1, HTT, NDUFA9 (includes EG:4704), NDUFS1, NDUFS2, NDUFS3, NDUFS5, NDUFV2, NUSAP1, PHB (includes EG:5245), PHLDA3, PTPRA, PURB, RGS16, RPS3A, SAAL1, SFN, TMEM25, TP53, TP63, TUBA4A, VDAC3, XPO5, YWHAG*	28	15	Organismal Development, Cell Cycle, Gene Expression
3	*ADAM2, ADAM3, ADAM1A, ALB, AMT (includes EG:275), ANKZF1, BCKDHA, C1ORF50, CAV1, CCT8, CTDSPL2, HDAC3, HNF1A, HNF4A, ITGA9, LMO3, MDFI (includes EG:4188), MIR34A (includes EG:407040), MRPL2, MYST2, NVL, ONECUT1, PCDHA12, PCIF1, PROX1, RORA, RXRA, SEC23A, SEC23IP, SLC25A13, SLC25A20, STAT3, ZNF193, ZNF225, ZNF324*	27	14	Cell-To-Cell Signaling and Interaction, Cellular Assembly and Organization, Reproductive System Development and Function
4	*APP, ASCL1, CD59, CNR1, Cytochrome c oxidase, DPYSL2, DPYSL3, DPYSL5, FCAR, FES, GARS, INS, KRT72, LPXN, MAP2K1/2, MAPK9, melatonin, NCF2, NCF1C, NSMAF, RAGE, RAPGEF3, REST, retinoic acid, RHOQ, Rsk, SLC26A4, SST, Tnf receptor, TRAF2, UBA1, UNC13B, WDR62, ZNF426, ZNF646*	23	13	Cell-To-Cell Signaling and Interaction, Hematological System Development and Function, Immune Cell Trafficking
5	*AGT, aldosterone, ATP6V0A4, BACH1, Calmodulin, CASP3, CD40LG, CD79B, CLEC4C, CNR1, CXCR4, cyclic AMP, FES, HMCN1 (includes EG:83872), IFNA2, IL4, IL15, IL12A, MAP6, MC1R, melatonin, NCF2, NCF1C, NDUFS1, NFATC2, OPRM1, PARG, PARP12, REN2, RGS2, RGS16, SPIN1, SST, TBX21, VDAC3*	19	11	Inflammatory Response, Cell-To-Cell Signaling and Interaction, Hematological System Development and Function
6	*ACTB, Actin, AKT1, amino acids, AURKC, BIRC5, CDK1, CDK4, DOK6, EGF, ERBB2, FER (includes EG:2241), GH1, IGF1, IGF1R, IRS1, JAK2, MAP3K1, MAPK13, MDM2, Plexin B, PLXNB2, POP1, PPHLN1, PPL, PPP2CA, PPP2R1A, PRKCA, PRKCE, PTK2B, RET, RPP14, SP1, SRC, VIM*	7	5	Amino Acid Metabolism, Post-Translational Modification, Small Molecule Biochemistry

Frontal cortex

ID	Molecules in Network	Score	Focus Molecules	Top Functions
1	*ADIPOR1, APP, AR, BBS2, BHMT, D-glucose, DLST, EGF, FSH, GH1, HNF4A, hydrogen peroxide, IGF1, Insulin, L-triiodothyronine, Laminin1, LARP1B (includes EG:55132), LMOD1, MDH2, MGST1, MTUS1, NPY, OGDH, PET112L, PLSCR1, POLR3E, POLRMT, PPARGC1A, SYTL3, TBCK, TMEM87B, UQCC, VEGFA, ZNHIT3*	29	15	Lipid Metabolism, Molecular Transport, Small Molecule Biochemistry
2	*Alpha catenin, ARHGAP5, C11ORF17, DYNLL1, EGF, GAST, GCC2, glucosamine, hydrogen peroxide, IGF1, Insulin, Jnk, KIAA0182, MAPK13, MDH2, MORC4, MYOG, PIK3R1, PLSCR1, Ponsin-Afadin-Nectin2, PPARGC1A, PPFIA3 (includes EG:8541), PRAM1, PTPN20B, PVRL2, SAE1, SKIL, SLC2A4, TGFB1, TTF2, USP25, USP28, VEGFA, YWHAZ*	24	12	Carbohydrate Metabolism, Cellular Development, Embryonic Development
3	*ADIPOR1, APP, BIRC2, Calmodulin, CARD8, Caspase, CDC23 (includes EG:8697), Ciap, D-glucose, EGF, FSH, glycochenodeoxycholate, GP5, GSN, GSN-PI3K-PIP2-Src, GSPT1, HTT, IGF1, IL16, Il15r, Insulin, MIR124, MYLK3, NFkB (complex), PCNT, PI3K, RHOB, SLC2A4, STK10, TARBP1 (includes EG:6894), TSC22D4, VEGFA, XAF1, XIAP/CIAP, ZMYND11*	22	12	Amino Acid Metabolism, Molecular Transport, Small Molecule Biochemistry
4	*ADCY, Alp, AP1G1, APP, ATP6V0A2, BDNF, beta-estradiol, BMP2, CAMKK1, COCH, cyclic AMP, DYNLL1, EGF, FBN1, GAS7, GFRA1, GH1, IGF1, IL10, Insulin, MIR17 (includes EG:406952), MYOG, NCKAP1L, NELL1, NOS2, PDE1B, retinoic acid, SFPQ, SLC12A5, SLC2A4, SOSTDC1, SYNRG, TG, VEGFA, ZNF804A*	22	13	Tissue Development, Cellular Development, Hematopoiesis
5	*ADCY, APP, CAPNS1, Cdc2, COL4A1, CRYM, cyclic AMP, D-glucose, DLG4, EDN1, EGF, FBLN5, FSH, GH1, hydrogen peroxide, IGF1, IL-2R, IL2RG, Insulin, ITGA10, JUN, L-triiodothyronine, Laminin1, LGALS3, MPRIP, MRPL12, MTHFR, MYC, MYCBP2, Pkg, PPP1R12A, sucrose, TG, VEGFA*	8	6	Developmental Disorder, DNA Replication, Recombination, and Repair, Cellular Growth and Proliferation

Parietal cortex

ID	Molecules in Network	Score	Focus Molecules	Top Functions
1	*ACTN1, ADD2, ANXA5, ARHGDIG, ATP2A3, C9, CAPZA2, Caspase, CFB, CLIP2, DDB2, ERK1/2, GSTM2, GSTP1, HMMR, Insulin, Interferon alpha, Jnk, Lh, LIMK2, NAIP, NARS, NFkB (complex), Pkc(s), PPP2R1A, PRDX1, PRKCI, PTGDR, PXN, RAB1A, RAP1GDS1, Ras homolog, SMC1B, TSHR, UBE2L6*	57	26	Lipid Metabolism, Small Molecule Biochemistry, Cellular Assembly and Organization
2	*ABCB9, ATP6V1D, C21ORF56, C9ORF5, CCDC59, CDK5, CETN2, CP110, CYP3A43, DLST, DPH5, EIF2AK2, GAPDH (includes EG:2597), HIST1H2BD, HNF4A, LGALS7B, LSG1, MAP2K3, MAP2K4, MAP3K3, MAPK14, MYL6, NDUFS1, NDUFS3, NDUFS4, OGDH, PAK7, PHB (includes EG:5245), PRKCE, S100A9, SLC25A5, TBC1D16, TRAF2, UMPS, VKORC1*	28	15	Free Radical Scavenging, Cardiovascular Disease, Skeletal and Muscular Disorders
3	*ACTN1, ALG1, amino acids, ATP6V0A2, BACE1, BBC3, CASP9, CAT, CSNK1E, CYC1, FSH, GLI1, GZMH, HCFC1, IL10, ILK, mannitol, OTUD4, PCNA, PDHB, PDHX, PDK2, PKN1, POLQ, PPP2R2A, PPP2R5C, PTPN4, PTPRC, SERPINF2, Sod, SP1, STK24, TGFB1, TNKS1BP1, TOB1*	23	13	Amino Acid Metabolism, Post-Translational Modification, Small Molecule Biochemistry
4	*ADORA2A, CTNNB1, CXCR2, DDX4, GSTM2, HBE1, HBG1, HBG2, HBZ, HOXA9, HSPD1, IL13, MAGI1, NCAN, NR2C2, OSM, PLEC, Ppp2c, PSMB8, PSMB9, psychosine, PTPRC, QPCT (includes EG:25797), RNF8, RXRA, SEPT11, SFMBT1, SNAP91, TADA3, TH, TNF, TNR, UGCG, VASH1, ZNF467*	22	13	Cell-To-Cell Signaling and Interaction, Tissue Development, Gene Expression
5	*ACTB, ACTN1, Ap1, BBC3, CSNK2B, EEA1, EGF, FBXO2, GH1, HCG 25371, IDH3A, IDH3G, LPHN2, MLH1, MYC, ND3, NKX3-2, NPHS1, NPHS2, PPP2R3A, PRDX1, PSMA3, PSMB3, PYGB, RAB5B, RAB7A, RIN1, RNF115, RTN1, Shc, SMARCB1, SNPH, TH, thyroid hormone, TXNIP*	21	12	Cellular Development, Cell Cycle, Connective Tissue Development and Function
6	*ACTB, ADAMTS5, ANXA2, AP2A2, ATP5B, BFSP2 (includes EG:8419), CAPN2, Caveolin, CD28, DCTN2, EIF4EBP1, F Actin, FGF1, FMR1, GCG, GPM6B, HRAS, HSPD1, HTT, KIF2B, LRMP, MIR302A (includes EG:407028), MIR34A (includes EG:407040), NAPG, NEGR1, NSF, PPP1R16B, RBPMS2, SLC25A22, Snare, SPTBN2, SRF, STAU1, TGM2, ZFYVE26*	20	12	Cellular Assembly and Organization, Cellular Compromise, Infection Mechanism
7	*19S proteasome, 26s Proteasome, ADRM1, AR, ATXN1, BRCA2, CDK4, COIL, CSNK2B, CYB5R2, PARK2, PLEKHG4, PSMA1, PSMA2, PSMA4, PSMA6, PSMA7, PSMB1, PSMB5, PSMB6, PSMB7, PSMB8, PSMB9, PSMB10, PSMC4, PSMD5, PSMD7, PSMD9, PSMD13, PSME1, SMAD2, SNCAIP, TRAF2, TRIP6, VCP*	7	5	Cellular Assembly and Organization, Cell Cycle, Cell-mediated Immune Response

Occpital cortex

ID	Molecules in Network	Score	Focus Molecules	Top Functions
1	*20s proteasome, 26s Proteasome, ACPP, C4BPB, CHAT, Ck2, Creb, DDX42, DSPP, DZIP3, ERBB2, G6PD, Histone h3, Histone h4, HOXB7, Hsp70, HTT, Laminin, MAGI2, MIR1, NFkB (complex), NID1, NKIRAS1, NUB1, PARK7, PI3K, POLR2A, PSMA8, PSMB6, RAB11FIP2, RIPK3, RNA polymerase II, RPS6KA1, SH2D4A, WDR36 (includes EG:134430)*	47	23	Genetic Disorder, Nervous System Development and Function, Skeletal and Muscular Disorders
2	*amino acids, ANXA3, ASS1, beta-estradiol, CDK2AP1, DSCAM, EEA1, ERH, FMO2, FOLH1, GNL2, HDAC1, HLTF, HMGCLL1, HUNK, IKZF1, IL4, IL5, IL15, ISG15, ITGAD, MBP, MIR98 (includes EG:407054), NR2E3, PDE4B, POLG, PTPN5, SET, SRM, TRIM28, UGT1A10 (includes EG:54575), ULK1, VIPR2, WDR44, ZFP53*	27	15	Lymphoid Tissue Structure and Development, Tissue Morphology, Cellular Development
3	*ABCD2, ARHGEF2, ATP5J2, CEP250, COPG, CSF3, DKC1, FAHD1, FGFBP2, LONP1, MAGED1, MBP, MME, MYC, NDUFA8, NDUFA9 (includes EG:4704), NDUFS2, NDUFS3, NFYB, PAK2, PIWIL1, PRKCD, PSMA1, PSMA2, PSMA3, PSMB7, PSMD6, RFX2, SLC27A6, TRIM41, TTLL9, UBR2, VDAC2, VIM, ZNF473*	27	15	Cellular Assembly and Organization, Cell-To-Cell Signaling and Interaction, Cellular Function and Maintenance
4	*ASCC2, ATL2, BMPR2, CCDC67, CCDC89, CHCHD3, CHCHD6, CYFIP1, DNAJC11, GIGYF2, HNRNPD, HTT, IFIT3, IMMT, ISG15, LANCL1, LDHAL6A, LDHAL6B, LDHC, LYST, MID1, MTX2, NDUFS3, OC90, retinoic acid, SERPINA3K, SP6, SPI1, SRGAP1, SRGAP2, SRRT, TXNDC11, VGLL3, YWHAZ, ZNF133*	25	14	Tissue Development, Cellular Development, Cellular Compromise
5	*ADAM15, AFF1, butyric acid, CDC42EP5, CLCN6, CNTN3, CYP1A1, DHRS1, DLG4, DPP6, DYNC2H1, FBN2 (includes EG:2201), FYN, GLI3, KCND2, KRAS, LRP8, MAN2A1, MAPK9, MBP, MDFI (includes EG:4188), MIR122 (includes EG:406906), MIR31 (includes EG:407035), MYOG, NRG1, OTOA, PAK2, RAB20, SEPT2, SEPT5, SEPT7, SEPT11, SYNGAP1, VLDLR, ZBTB25*	22	13	Nervous System Development and Function, Neurological Disease, Cell-To-Cell Signaling and Interaction
6	*ABCC3, AGGF1, ALPL, ASCC3, BECN1, CAR ligand-CAR-Retinoic acid-RXRα, CENPI, COMMD8, COMMD1 (includes EG:150684), CX3CL1, ELL2, FAF1, FSH, GAD1, LEP, lithocholic acid, MED15, NR1I2, OPLAH, PPP2R1A, PXR ligand-PXR-Retinoic acid-RXRα, RELA, RGS16, SCP2, SKIL, SLCO1A2, SMAD1, STAT6, TNFSF12, ursodeoxycholic acid, USP25, ZEB2, ZSCAN4, ZZEF1*	16	10	Lipid Metabolism, Molecular Transport, Small Molecule Biochemistry
7	*Akt, BLM, CHCHD2, CNP, CNTF, creatine, ERK1/2, GDNF, Glycogen synthase, GUCY2C, hCG, Insulin, Jnk, MAP3K3, Mapk, MBP, MCAM, mevalonic acid, OPRK1, P38 MAPK, PAK2, PCSK9, PEA15, Pi3-kinase, PIK3R3, PYGM, Ras, RGS20, S100A1, SET, SOS1, SP100, STXBP4, TRIM63, TYRO3*	12	8	Skeletal and Muscular Disorders, Organismal Injury and Abnormalities, Cell Death

Amygdala

ID	Molecules in Network	Score	Focus Molecules	Top Functions
1	*ACACA, Actin, ADAM19, alcohol group acceptor phosphotransferase, BLM, CCR1, CDK18, Collagen type I, Collagen type IV, ERK1/2, FMNL1, Focal adhesion kinase, G protein alpha, Gpcr, GRK4, hCG, Interferon alpha, LPAR2, MAPK1, MAPKAPK2, Metalloprotease, MTTP, NOS3, PDGF BB, PFN2, Pkc(s), PLC, PP2A, PPP2R4, PRKD3, PRKG2, Rock, SREBF2, T3-TR-RXR, TPM3*	34	18	Cell Cycle, Cardiovascular System Development and Function, Organ Development
2	*Ap1, CALB1, CD7, CSDE1, CXCL12, EGFR, Estrogen Receptor, Fibrinogen, FKBP1A, G protein alphai, GH1, Growth hormone, HBB (includes EG:3043), HDL, HIVEP1, Insulin, LDL, LPL, Mapk, MBL2, Mek, NFkB (complex), NRG, Pi3-kinase, PTPN1, Rac, Rap1, Ras, Sapk, SCGB1A1, SH3BP1, TNFRSF10A (includes EG:8797), UTRN, VAV, Vegf*	28	16	Cellular Movement, Tumor Morphology, Cell Morphology
3	*amino acids, ARHGEF1, BMP7, CCDC85B, CDH1, DNAH2, DUSP13, ERBB2, GBAS, HCK, HOXA13, Laminin1, LNX1, LYN, MAOA, MUC16, MYO18A, NIPSNAP1, NPHS1, NUDT3, PRKAR1A, PRKCI, progesterone, RANBP6, RBM12, SFRP1, SHROOM3, SMAD2, SPAG1, TGFB1, TPM1, TPM2, TPM3, YLPM1, ZNF337*	24	14	Cell-To-Cell Signaling and Interaction, Cellular Function and Maintenance, Cancer
4	*ALB, ALDH16A1, beta-estradiol, BMP4, CALB1, CCNG1, CEP152, EPB41L3, HELLS, HNF1A, HNF1B, HRAS, IKBKB, KAT2B, KCNMA1, KLF1, lipid, MYH4, MYH10, MYH14, OBSL1, PKHD1, PRDX6, RPL37, SFXN5, SLC22A12, SOD2, SYP, TAF6L, TMOD2, Tropomyosin, TRPM6, TSC22D1, TWSG1, ZNF526*	22	13	Cancer, Cell Death, Cellular Development
5	*ABCF2, ABL1, ALDH1A1, ANKZF1, BDNF, Calmodulin, CEBPB, CLPP, CYP1A1, DKK1, DYNC2LI1, GNPAT, HNF4A, HP, INS1, ISL1, KLF12, LDB1, LMX1A, NDUFA4, NRSN2, OTC, PRKCI, PRKCZ, RBL2, RNA polymerase II, RRP8, SFRP1, SNAP23, STK11, SUV39H1, TAOK3, TTYH1, ZFP106, ZKSCAN5*	22	13	Cellular Development, Hepatic System Development and Function, Lipid Metabolism
6	*AMMECR1, ATP11A, CCT4, COX2, COX3, COX4I1, COX5B, CUL1, CYTB, Cytochrome c oxidase, DMRTB1, E2F1, GABPB1 (includes EG:2553), HNRNPA1, IFNB1, IKBKB, IKBKE, IL16, INO80C, KCTD13, LPPR4, MIR122 (includes EG:406906), MIR183 (includes EG:406959), NQO2, PELI1, PGRMC1, SAT1, SFXN1, TNF, TNFSF12, TPM3, UBR2, UBXN7, UQCRFS1, VPS54*	20	12	Inflammatory Disease, Inflammatory Response, Cell Death
7	*Akt, ATP6V0D1, Calmodulin, Ck2, Creb, CREBZF, DNAJC5, E2f, ERK, ETV5, FSH, G protein beta gamma, Histone h3, Hsp70, Hsp90, IgG, IL1, Jnk, KLF5, Lh, MYBL2, NPHS1, P38 MAPK, PI3K, Pka, Ras homolog, RNA polymerase II, SMARCB1, SMC4, SNCA, STUB1, TPX2, TXLNA (includes EG:200081), UNC13B, VAMP2*	18	11	Molecular Transport, Small Molecule Biochemistry, Cancer
8	*20s proteasome, ABL1, ATP5B, BAI3, CAV1, COX2, COX3, COX17, COX5B, COX6B1, CTSB, Cytochrome c oxidase, FAM173A, GAPVD1, HSPA8, HTT, IGSF9B, IL3, iron, KAT2B, MEIS2, MIR141 (includes EG:406933), MIR181B2, PHLPP2, PPP2CA, PRKCG, SFXN3, SIAH1, SLC25A3, SNCA, SUMO1, TGM2, TP53, UCHL1, YWHAG*	10	7	Neurological Disease, Cell Death, Nervous System Development and Function
