# Supplementary figures and images for: Proteomic analysis of proteins expressing in regions of rat brain by a combination of SDS-PAGE with nano-liquid chromatography-quadrupole-time of flight tandem mass spectrometry
Source: Proteome Sci. 2010 Jul 27;8:41. doi: 10.1186/1477-5956-8-41 (PMC2918549; doi:10.1186/1477-5956-8-41)

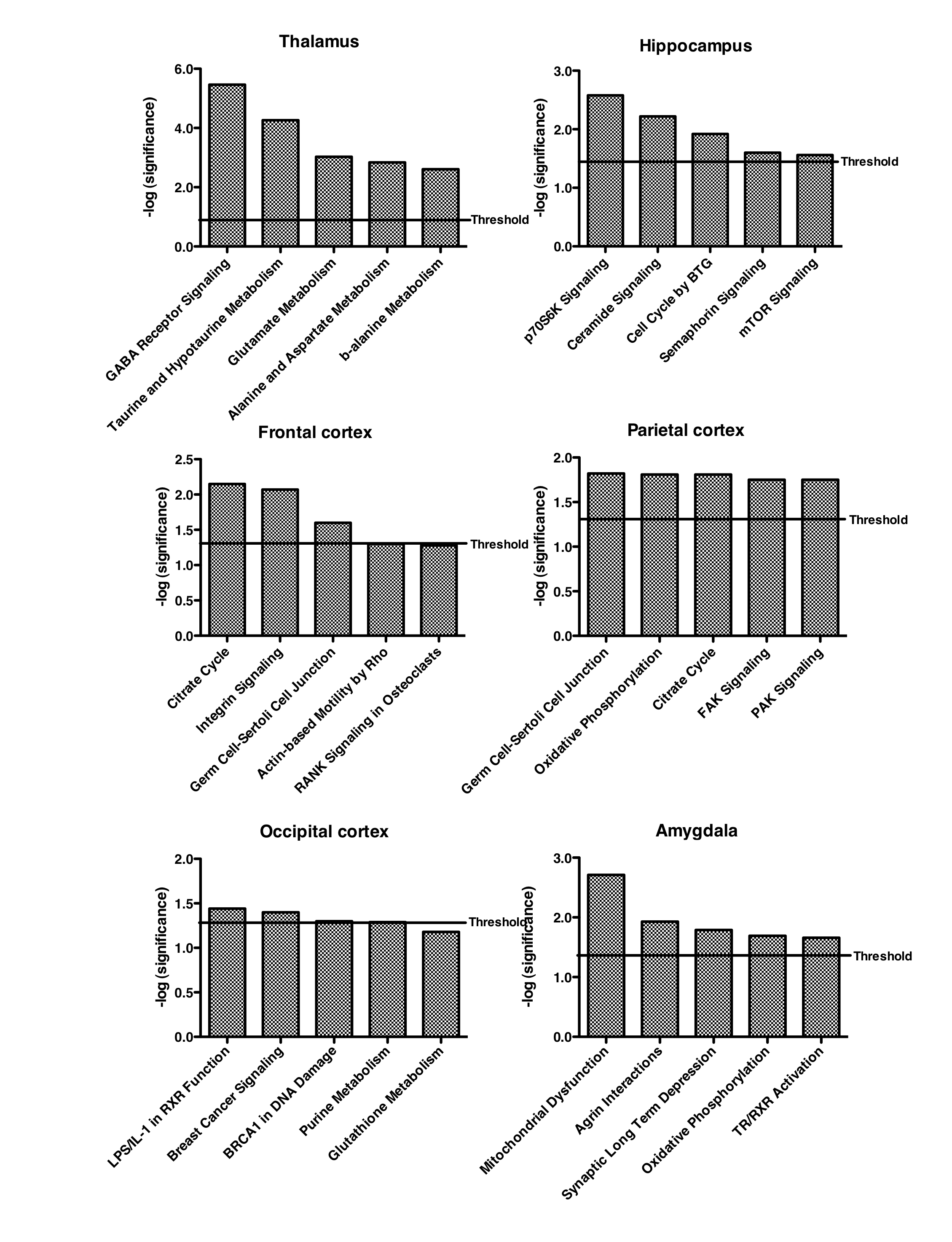

Supplement: Additional file 3 — Figure S1. Canonical pathways analyzed by proteins identified in each region of rat brain. [file 1477-5956-8-41-S3.TIFF]
